# Rates of Dinosaur Body Mass Evolution Indicate 170 Million Years of Sustained Ecological Innovation on the Avian Stem Lineage

**DOI:** 10.1371/journal.pbio.1001853

**Published:** 2014-05-06

**Authors:** Roger B. J. Benson, Nicolás E. Campione, Matthew T. Carrano, Philip D. Mannion, Corwin Sullivan, Paul Upchurch, David C. Evans

**Affiliations:** 1 Department of Earth Sciences, University of Oxford, Oxford, United Kingdom; 2 Departments of Earth Sciences (Palaeobiology) and Organismal Biology (Evolution and Development), Uppsala University, Uppsala, Sweden; 3 Department of Ecology and Evolutionary Biology, University of Toronto, Toronto, Canada; 4 Department of Paleobiology, Smithsonian Institution, Washington DC, United States of America; 5 Department of Earth Science and Engineering, Imperial College London, London, United Kingdom; 6 Key Laboratory of Vertebrate Evolution and Human Origins, Institute of Vertebrate Paleontology and Paleoanthropology, Beijing, China; 7 Department of Earth Sciences, University College London, London, United Kingdom; 8 Department of Natural History, Royal Ontario Museum, Toronto, Canada; Ecole Normale Supérieure, France

## Abstract

Large-scale adaptive radiations might explain the runaway success of a minority of extant vertebrate clades. This hypothesis predicts, among other things, rapid rates of morphological evolution during the early history of major groups, as lineages invade disparate ecological niches. However, few studies of adaptive radiation have included deep time data, so the links between extant diversity and major extinct radiations are unclear. The intensively studied Mesozoic dinosaur record provides a model system for such investigation, representing an ecologically diverse group that dominated terrestrial ecosystems for 170 million years. Furthermore, with 10,000 species, extant dinosaurs (birds) are the most speciose living tetrapod clade. We assembled composite trees of 614–622 Mesozoic dinosaurs/birds, and a comprehensive body mass dataset using the scaling relationship of limb bone robustness. Maximum-likelihood modelling and the node height test reveal rapid evolutionary rates and a predominance of rapid shifts among size classes in early (Triassic) dinosaurs. This indicates an early burst niche-filling pattern and contrasts with previous studies that favoured gradualistic rates. Subsequently, rates declined in most lineages, which rarely exploited new ecological niches. However, feathered maniraptoran dinosaurs (including Mesozoic birds) sustained rapid evolution from at least the Middle Jurassic, suggesting that these taxa evaded the effects of niche saturation. This indicates that a long evolutionary history of continuing ecological innovation paved the way for a second great radiation of dinosaurs, in birds. We therefore demonstrate links between the predominantly extinct deep time adaptive radiation of non-avian dinosaurs and the phenomenal diversification of birds, via continuing rapid rates of evolution along the phylogenetic stem lineage. This raises the possibility that the uneven distribution of biodiversity results not just from large-scale extrapolation of the process of adaptive radiation in a few extant clades, but also from the maintenance of evolvability on vast time scales across the history of life, in key lineages.

## Abstract 2

Early dinosaurs showed rapid evolutionary rates, which were sustained on the line leading to birds. Maintenance of evolvability in key lineages might explain the uneven distribution of trait diversity among groups of animal species.

## Author Summary

Animals display huge morphological and ecological diversity. One possible explanation of how this diversity evolved is the "niche filling" model of adaptive radiation—under which evolutionary rates are highest early in the evolution of a group, as lineages diversify to fill disparate ecological niches. We studied patterns of body size evolution in dinosaurs and birds to test this model, and to explore the links between modern day diversity and major extinct radiations. We found rapid evolutionary rates in early dinosaur evolution, beginning more than 200 million years ago, as dinosaur body sizes diversified rapidly to fill new ecological niches, including herbivory. High rates were maintained only on the evolutionary line leading to birds, which continued to produce new ecological diversity not seen in other dinosaurs. Small body size might have been key to maintaining evolutionary potential (evolvability) in birds, which broke the lower body size limit of about 1 kg seen in other dinosaurs. Our results suggest that the maintenance of evolvability in only some lineages explains the unbalanced distribution of morphological and ecological diversity seen among groups of animals, both extinct and extant. Important living groups such as birds might therefore result from sustained, rapid evolutionary rates over timescales of hundreds of millions of years.

## Introduction

Much of extant biodiversity may have arisen from a small number of adaptive radiations occurring on large spatiotemporal scales [1]–[3]. Under the niche-filling model of adaptive radiation, ecological opportunities arise from key innovations, the extinction of competitors, or geographic dispersal [1],[4],[5]. These cause rapid evolutionary rates in ecologically relevant traits, as diverging lineages exploit distinct resources. Rates of trait evolution then decelerate as niches become saturated, a pattern that has been formalised as the “early burst” model (e.g., [6],[7]).

Most phylogenetic studies of adaptive radiations focus on small scales such as island radiations and other recently diverging clades, including *Anolis* lizards, cichlid fishes, and geospizine finches [2],[6],[8]–[10]. Detailed study of these model systems has demonstrated the importance of ecological and functional divergence as drivers of speciation early in adaptive radiations (e.g., [11],[12]). Surprisingly though, early burst patterns of trait evolution receive only limited support from model comparison approaches for these and other adaptive radiations occurring in geographically restricted areas and on short timescales (<50 million years [Ma]; most <10 Ma) [6] (but see [13],[14]).

Studies of morphological evolution on longer timescales, unfolding over 100 Ma or more, are central to establishing whether niche-filling or early burst patterns of trait evolution are important evolutionary phenomena on large phylogenetic scales. A small number of recent studies quantified patterns of trait evolution on large scales using neontological phylogenies. For example, diversification rates and morphological rates are positively correlated in actinopterygians [15] (∼400 Ma); rapid rates of both morphological and molecular evolution occur on deep, Cambrian, nodes of the arthropod tree of life [16] (∼540 Ma); and the early evolution of placental mammals was characterised by rapid rates of diversification [17] (100–65 Ma) and perhaps body size evolution [18] (but see [19]).

However, even the largest neontological studies [15]–[18],[20],[21] are limited to explaining the rise of important extant groups. A more complete characterisation of macroevolutionary processes on long timescales should also explain the ascent and demise of important extinct groups (e.g., [22]), which in fact represent most of life's diversity. Substantial evidence for the dynamics of past adaptive radiations might have been erased from the neontological archive, and macroevolutionary models for extinct or declining/depauperate clades may be tested most effectively using deep time data from the fossil record [23],[24].

Palaeontologists often quantify patterns of morphological radiation using time series of disparity (e.g., [25],[26]). However, few phylogenetic studies including fossil data have attempted to explain patterns of morphological radiation in large clades on timescales >100 Ma, and most have individually targeted either the roots of exceptional modern clades such as birds or mammals (e.g., [19],[27],[28]) or extinct/depauperate clades (e.g., [29]–[31]; studies based on discrete characters). Thus, patterns of morphological evolution in major extinct clades, and their links to successful modern clades, are not well understood.

**Figure 1:**
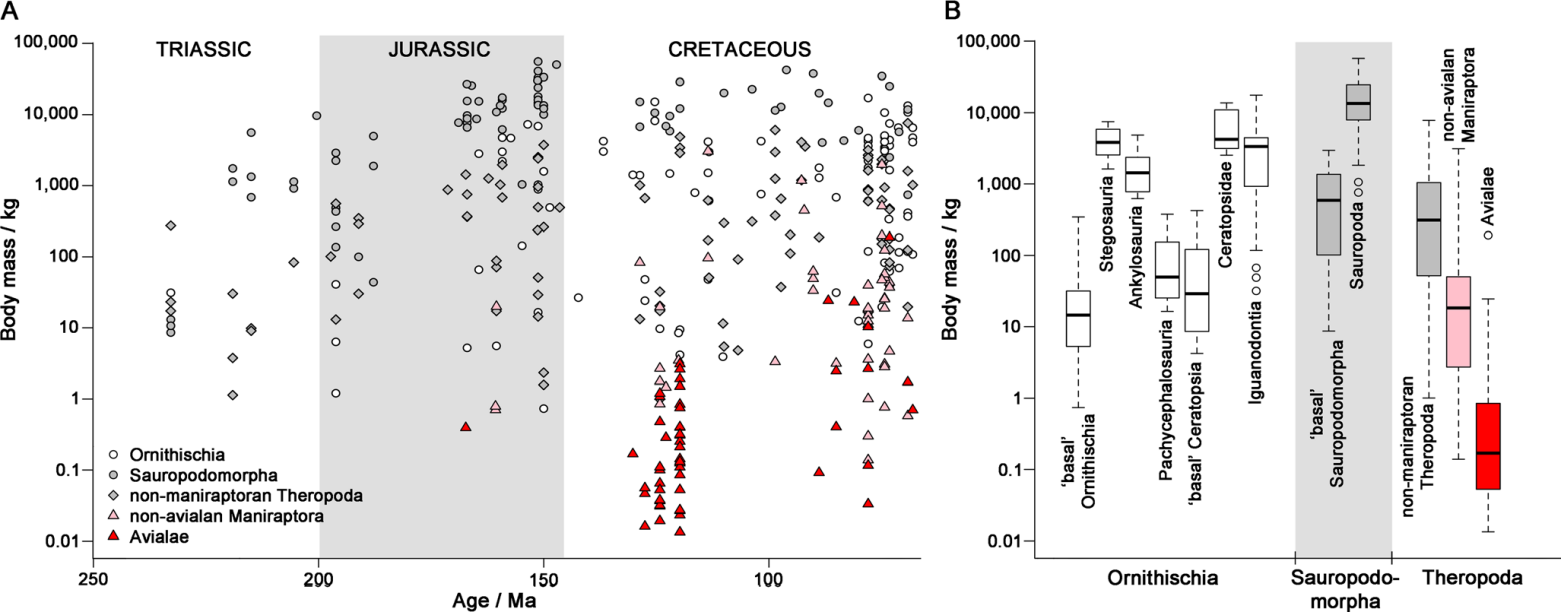
Dinosaur body masses. (A) Dinosaur body mass through time (the full set of mass estimates is given in Dataset S1). (B) Box-and-whisker plot showing median (dark line), hinges (box range), and ranges (whiskers) of body masses for major dinosaur groups. Outliers (circles) include the iguanodontians *Mochlodon vorosi* (31 kg), *Elrhazosaurus*, and *Valdosaurus* (both 48 kg), the sauropods *Europasaurus* (1,050 kg) and *Magyarosaurus* (746 kg), and the flightless avialan *Gargantuavis* (180 kg).

Non-avian dinosaurs are an iconic group of terrestrial animals. They were abundant and ecologically diverse for most of the Mesozoic, and included extremely large-bodied taxa that challenge our understanding of size limits in terrestrial animals [32]. The first dinosaurs appeared more than 230 Ma ago in the Triassic Period, as small-bodied (10–60 kg), bipedal, generalists. By the Early Jurassic (circa 200 Ma), they dominated terrestrial ecosystems in terms of species richness [33],[34], and Cretaceous dinosaurs (145–66 Ma) had body masses spanning more than seven orders of magnitude (Figure 1A). Non-avian dinosaurs became extinct at the catastrophic Cretaceous/Paleogene (K/Pg) boundary event, at or near the peak of their diversity [35],[36]. In contrast, extant dinosaurs (neornithine birds) comprise around 10,000 species and result from one of the most important large-scale adaptive radiations of the Cenozoic [3],[21].

The proposed drivers of early dinosaur diversification are controversial. Although various causal factors have been suggested to underlie a presumed adaptive radiation, few studies have tested the predictions of niche-filling models, and these have yielded equivocal results. An upright, bipedal gait, rapid growth, and possible endothermy have been proposed as key innovations of Triassic dinosaurs (reviewed by [34]), and mass extinctions during the Triassic/Jurassic boundary interval removed competing clades, perhaps leading to ecological release and rapid rates of body size evolution in Early Jurassic dinosaurs [37] (but see [34]). However, quantitative studies using body size proxies [34] and discrete morphological characters [33] have found only weak support for the niche-filling model during early dinosaur evolution, instead favouring gradualistic evolutionary rates. These studies focussed on the Late Triassic–Early Jurassic, so it is unclear whether Early Jurassic dinosaur evolution differed from later intervals (consistent with radiation following a mass extinction), or how the Middle Jurassic–Cretaceous radiation of birds and their proximate relatives relates to overall patterns of dinosaur diversification.

We used phylogenetic comparative methods [6],[14],[38],[39] to analyse rates of dinosaur body mass evolution (*Materials and Methods*; Appendix S1). For this study, we compiled a large dataset of dinosaur body masses (441 taxa; Dataset S1) using the accurate scaling relationship of limb robustness (shaft circumference) derived from extant tetrapods [40] (Appendix S1; Dataset S1). Body mass affects all aspects of organismal biology and ecology (e.g., [41],[42]), including that of dinosaurs (e.g., [43]–[45]). Because of its relationship with animal energetics and first-order ecology, understanding the evolution of body mass is fundamental to identifying the macroevolutionary processes underlying biodiversity seen in both ancient and modern biotas. Therefore, by studying body mass evolution, we assess the broad pattern of niche filling in the assembly of dinosaur diversity through 170 Ma of the Mesozoic.

In many hypotheses of adaptive radiation, ecological speciation is an important process generating both morphological and taxonomic diversity (e.g., [2]; but see [46]), according to which ecological differentiation is essentially simultaneous with lineage splitting [12]. In consequence, many large-scale studies of adaptive radiation have focussed on diversification rates (e.g., [17],[21],[47]). A correlation between diversification rates and morphological rates is consistent with adaptive radiation (e.g., [15]). However, even when this can be demonstrated, the occurrence of ecological speciation is difficult (perhaps impossible) to test in clades even only a few Ma old [48]. Methods for estimating diversification rates on non-ultrametric trees (e.g., those including deep time data) have recently become available [49]. However, these methods require accurate estimates of sampling probability during discrete time intervals, and it is not clear that it is possible to obtain such estimates from the dinosaur fossil record, which contains many taxa known only from single occurrences. Therefore, our study focuses on the predictions of niche-filling models of morphological evolution during adaptive radiation, as done in some previous studies (e.g., [6],[13]).

## Results

Most of the earliest dinosaurs weighed 10–35 kg (Figure 1); *Herrerasaurus* was exceptionally large at 260 kg. Maximum body masses increased rapidly to 1,000–10,000 kg in sauropodomorphs, with especially high masses in early sauropods such as *Antetonitrus* (5,600 kg; Norian, Late Triassic) and *Vulcanodon* (9,800 kg; Early Jurassic), whereas minimum body masses of 1–4 kg were attained by Late Triassic ornithischians and theropods (Figure 1). Jurassic Heterodontosauridae (∼0.7 kg [50]), Middle Jurassic and younger Paraves (e.g., Epidexipteryx, 0.4 kg; Anchiornis, 0.7 kg), and Cretaceous Avialae (birds: 13–16 g to 190 kg [51]) extended this lower body size limit (Table 1). *Archaeopteryx* weighed 0.99 kg (the largest, subadult specimen [52]) and the Cretaceous sauropod *Argentinosaurus* weighed approximately 90,000 kg (Table 1). Our full set of mass estimates is available in Dataset S1 and a summary is presented in Table 1.

**Table 1:** Estimated masses in kilograms of smaller- and larger-bodied adult representatives of major dinosaur groups, given to two significant figures. The standard error of all mass estimates is 0.135 log_10_(kg) [40].

Clade	Smaller masses		Larger masses	
**Theropoda**				
Theropoda (non-maniraptoran)	Sinosauropteryx prima	*0.99*	*Tyrannosaurus rex*	7,700
	*Procompsognathus triassicus*	1.13	*Giganotosaurus carolinii*	6,100
Maniraptora (non-avialan)	*Parvicursor remotus*	0.14	*Suzhousaurus megatherioides*	3,100
	*Rahonavis ostromi*	0.58	*Gigantoraptor erlianensis*	2,000
Avialae	*Qiliania graffini*	0.013	*Gargantuavis philoinos*	190
	*Iberomesornis romerali*	0.016	*Hesperornis crassipes*	24
**Sauropodomorpha**				
Basal Sauropodomorpha	*Pampadromaeus barberenai*	8.5	*Lufengosaurus magnus*	2,300
Sauropoda	*Magyarosaurus dacus*	750	*Argentinosaurus huinculensis* a	90,000
	*Europasaurus holgeri*	1,000	*Brachiosaurus altithorax*	56,000
	*Lirainosaurus astibiae*	1,800	*Turiasaurus riodevensis*	51,000
**Ornithischia**				
Heterodontosauridae	*Fruitadens haagarorum*	0.73		
	*Tianyulong confuciusi*	0.74		
Stegosauria	*Kentrosaurus aethiopicus*	1,600	*Dacentrurus armatus*	7,400
Ankylosauria	*Saichania chulsanensis*	610	*Ankylosaurus magniventris*	4,800
Pachycephalosauria	*Stegoceras validum*	16	*Pachycephalosaurus wyomingensis*	370
Basal Ceratopsia	*Psittacosaurus sinensis*	4.1	*Leptoceratops gracilis*	420
Ceratopsidae	*Centrosaurus apertus*	2500	*Triceratops horridus*	14,000
Basal Iguanodontia	*Mochlodon vorosi*	31	*Iguanodon bernissartensis*	15,000
Hadrosauroidea	*Gilmoreosaurus mongoliensis*	1,300	*Edmontosaurus regalis*	7,600
	“*Probactrosaurus*” *mazongshanensis*	1,500	*Shantungosaurus giganteus*	17,000

a Only a referred femur of *Argentinosaurus* is known: estimating its humeral circumference from the least-squares regression relationship between humeral and femoral circumferences for large sauropods (femoral circumferences >400 mm) yields a mass estimate of 67,400–124,000 kg (95% prediction interval).

**Figure 2:**
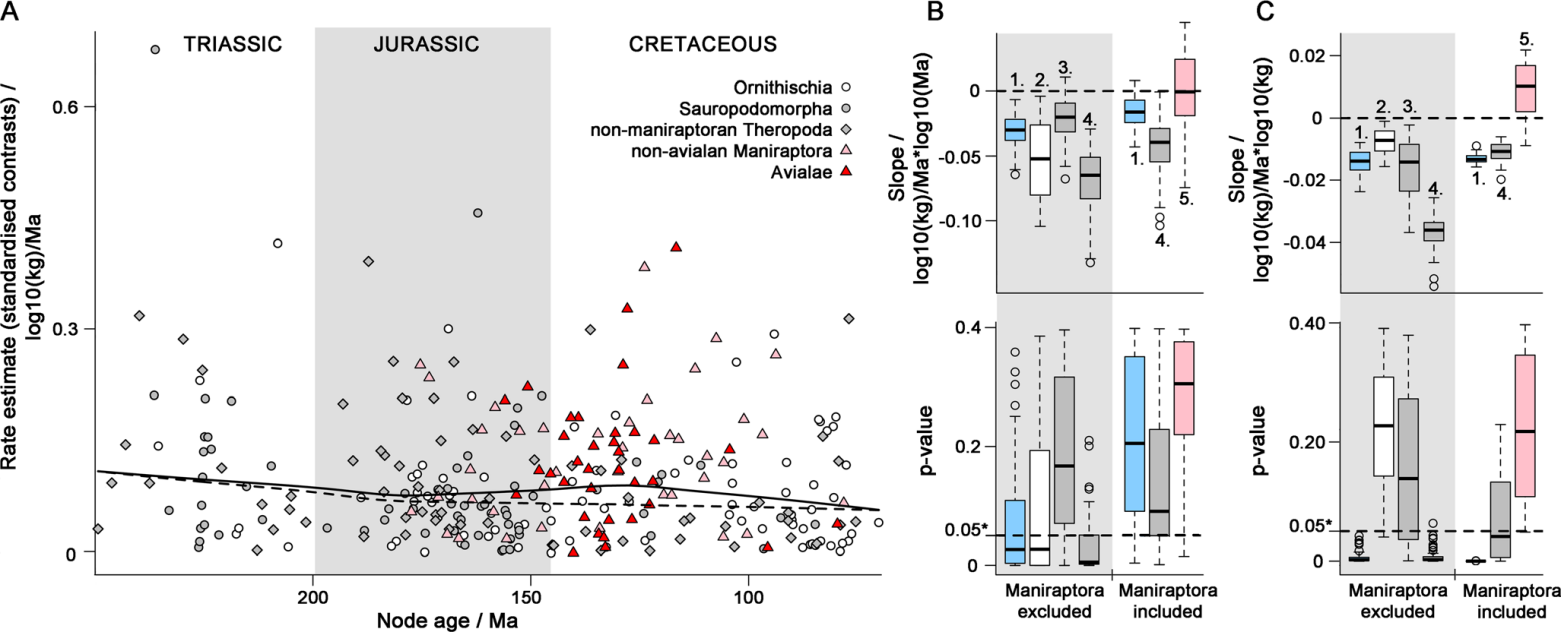
Node height test for early burst of rates of dinosaur body mass evolution. (A) Nodal evolutionary rate estimates (standardised independent contrasts [39],[89]) versus node age for data excluding (dashed lowess line) and including (solid lowess line) Maniraptora. (B–C) Box-and-whisker plots detailing results of: (B) robust regression of evolutionary rate on node age: slope (upper plot) and p-value (lower plot); (C) robust regression of evolutionary rate on nodal body mass: slope (upper plot) and p-value (lower plot). In (B–C) dashed lines occur at zero (upper plots) and 0.05 (lower plots: threshold for statistical significance). 1 = Dinosauria; 2 = Ornithischia; 3 = Sauropodomorpha; 4 = Theropoda; and 5 = Maniraptora.

Our node height tests indicate that evolutionary rate estimates at phylogenetic nodes (standardised phylogenetically independent contrasts [39]) vary inversely with log-transformed stratigraphic age for most phylogenies (Figure 2). This relationship is significant (based on robust regression [14],[53]) for most phylogenies of non-maniraptoran dinosaurs, and for ornithischians and non-maniraptoran theropods when analysed separately (Figure 2B). This result is weakened, and becomes non-significant, when Triassic nodes are excluded (Figure S1).

Declining evolutionary rates through time are not found in any analyses including maniraptorans. Indeed, when maniraptorans are added to analyses of Dinosauria, a burst of high nodal rate estimates is evident in lowess lines spanning the Middle Jurassic–Early Cretaceous interval of maniraptoran diversification (Figure 2A). Maniraptorans have a weakly positive (non-significant) relationship between evolutionary rates and body mass, and do not show diminishing evolutionary rates through time (Figure 2B–C). This contrasts with non-maniraptoran dinosaurs, in which evolutionary rates vary inversely with body mass (Figure 2C).

Maximum-likelihood models [6],[38] were fitted to phylogenies calibrated to stratigraphy using the “equal” and “mbl” (minimum branch length) methods (see *Materials and Methods*), and complement the results of our node height tests in showing support for early burst models only in analyses excluding Maniraptora (Table 2; Figure S2). Note, however, that the maximum-likelihood method has less statistical power to detect early burst patterns than does the node height test when even a small number of lineages escape from the overall pattern of declining rates through time [14]. Two models that predict saturation of trait variance through a clade's history were commonly supported in our analyses: the early burst model of exponentially declining evolutionary rates through time, and the Ornstein–Uhlenbeck (OU) model of attraction to a “trait optimum” value. Other models (e.g., Brownian motion, stasis) had negligible AICc weights in all or most (directional trend model) analyses (AICc is Akaike's information criterion for finite sample sizes).

**Table 2:** Summary of maximum-likelihood model-fitting approaches, AICc weights (see also Figure S2), and parameter values provided in the form “median (minimum–maximum)” over a set of 60 time-calibrated phylogenies (for AICc weights) or for those phylogenies in which the model received an AICc weight greater than 0.3 (the number of which is given in the column “Number”).

Early burst	AICc weight	Number (weight>0.3)	β_0_	a		
Dinosauria	0.0000 (0–0.004)	0	NA	NA		
Dinosauria (non-maniraptoran)	0.9615 (0–1)	33	0.043 (0.031–0.064)	−0.014 (−0.008–0.016)		
Ornithischia	0.6445 (0.158–0.999)	50	0.039 (0.020–0.057)	−0.010 (−0.005–0.017)		
Sauropodomorpha	0.6945 (0.002–1)	46	0.033 (0.016–0.081)	−0.017 (−0.005–0.017)		
Theropoda	0.0000 (0–0)	0	NA	NA		
Theropoda (non-maniraptoran)	0.7745 (0.048–0.999)	47	0.049 (0.033–0.085)	−0.014 (−0.011–0.021)		
Maniraptora	0.0000 (0–0.0450)	0	NA	NA		

Trend	AICc weight	Number (weight>0.3)	β	μ		
Dinosauria	0 (0–0)	0	NA	NA		
Dinosauria (non-maniraptoran)	0 (0–0.007)	0	NA	NA		
Ornithischia	0.1065 (0.001–0.357)	2	0.016 (0.016–0.016)	0.006 (0.006–0.006)		
Sauropodomorpha	0.001 (0–0.080)	0	NA	NA		
Theropoda	0 (0–0)	0	NA	NA		
Theropoda (non-maniraptoran)	0.008 (0–0.051)	0	NA	NA		
Maniraptora	0.001 (0–0.430)	1	0.019	0.011		

Ornstein–Uhlenbeck	AICc weight	Number (weight>0.3)	β	α	Z_0_	θ
Dinosauria	1.000 (0.996–1)	60	0.032 (0.022–0.045)	0.027 (0.016–0.047)	1.207 (−0.181–2.464)	2.349 (2.202–2.513)
Dinosauria (non-maniraptoran)	0.037 (0–1)	29	0.025 (0.021–0.029)	0.020 (0.014–0.027)	1.370 (1.007–1.859)	2.722 (2.571–2.838)
Ornithischia	0.036 (0–0.486)	9	0.020 (0.020–0.023)	0.010 (0.007–0.012)	1.383 (1.296–1.405)	2.453 (2.339–2.821)
Sauropodomorpha	0.733 (0–1)	43	0.025 (0.016–0.058)	0.041 (0.017–0.116)	0.328 (−3.871–1.118)	3.658 (3.283–4.305)
Theropoda	1 (1–1)	60	0.0465 (0.030–0.064)	0.039 (0.025–0.053)	1.434 (0.892–1.987)	1.923 (1.687–2.181)
Theropoda (non-maniraptoran)	0.189 (0.001–0.940)	27	0.039 (0.028–0.046)	0.037 (0.026–0.044)	1.417 (1.095–1.974)	2.342 (2.226–2.472)
Maniraptora	0.998 (0.451–1)	60	0.051 (0.024–0.078)	0.029 (0.009–0.051)	0.357 (−0.067–1.128)	0.907 (0.637–1.348)

Parameters: **β**, Brownian variance (log_10_kg^2^/Ma) (∼evolutionary rate; stochastic rate for Ornstein–Uhlenbeck [OU] models; initial rate [**β_0_**] in early burst models); **a**, a parameter describing variation in evolutionary rates through time in early burst models; **μ**, the mean step length (log_10_kg/Ma), indicating directional evolution in trend models; **α**, the strength of attraction to a macroevolutionary optimum (θ) in OU models; **Z_0_**, the ancestral node value (log_10_kg) in OU models; **θ**, the macroevolutionary optimum (log_10_kg) in OU models.

Early burst models received high AICc weights for analyses of ornithischians, non-maniraptoran theropods, and non-maniraptoran dinosaurs when using the “equal” branch length calibration method (Table 2; Figure S2). Early burst models had comparable AICc weights to Ornstein–Uhlenbeck models for sauropodomorphs when using the “equal” branch length calibration method, and for ornithischians and non-maniraptoran theropods when using the “mbl” method. Early burst models had generally lower AICc weights for non-maniraptoran dinosaurs and for sauropodomorphs when using the “mbl” branch length calibration method (Table 2; Figure S2). Support from some phylogenies for Ornstein–Uhlenbeck models of attraction to a large body size optimum from small ancestral body sizes [54],[55] in ornithischians [56], non-maniraptoran theropods, and especially sauropodomorphs and non-maniraptoran dinosaurs (Table 2; Figure S2), suggests the occurrence of Cope's rule in dinosaurs. All phylogenies provide strong support for this pattern in maniraptorans (Table 2).

**Figure 3:**
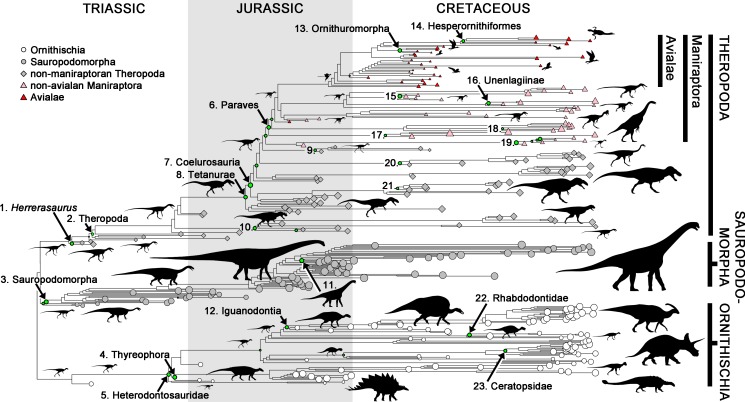
Dinosaur phylogeny showing nodes with exceptional rates of body size evolution. Exceptional nodes are numbered and indicated by green filled circles with diameter proportional to their down-weighting in robust regression analyses (Appendix S1). Details of these nodes are given in Table 2. The sizes of shapes at tree tips are proportional to log_10_(mass), and silhouettes are indicative of approximate relative size within some clades. The result from one tree calibrated to stratigraphy by imposing a minimum branch duration of 1 Ma is shown; other trees and calibration methods retrieve similar results. Silhouettes used were either previously available under Public Domain or with permission from the artists. Non-avialan dinosaur silhouettes used with thanks to the artist, Scott Hartman. Avialan silhouettes are modified from work by Nobumichi Tamura, and /Archaeopteryx/ from Mike Keesey.

Exceptionally high rates at individual nodes in our phylogenies were identified as down-weighted datapoints in robust regression analyses [14],[53]. Five sets of exceptional nodes in the Triassic–Early Jurassic represent rapid evolutionary shifts from primitive masses around 10–35 kg to large body masses in derived sauropodomorphs (>1,000 kg), armoured ornithischians (Thyreophora; Figure 1B) and theropods (*Herrerasaurus*, and derived taxa such as *Liliensternus* (84 kg) and *Dilophosaurus* (350 kg)), and to smaller body sizes in heterodontosaurid ornithischians (Figure 3; Table 3). Rapid body size changes were rare in later ornithischians and sauropodomorphs, which each show only one exceptional Jurassic node, marking the origin of body sizes greater than 1,000 kg in derived iguanodontians, and of island dwarfism in the sauropod *Europasaurus* [57]. By contrast, up to six exceptional Jurassic nodes occur in theropod evolution, with especially high contrasts at the origins of body sizes exceeding 750 kg in Tetanurae, and marking phylogenetically nested size reductions on the line leading to birds: in Coelurosauria (e.g., *Ornitholestes*, 14 kg; *Zuolong*, 88 kg) and in Paraves, which originated at very small body masses around 1 kg [58].

**Table 3:** Details of body size changes at exceptional nodes indicated in Figure 3.

Node	Description	Clade	Date	Polarity	Hypothesis
1	Origin of large body size in the early theropod *Herrerasaurus* (260 kg)	Thero.	Triassic	Increase	Macropredation
2	Origin of large body size in derived theropods such as *Liliensternus* (84 kg) and *Dilophosaurus* (350 kg)	Thero.	Triassic	Increase	Macropredation
3	Origin of large body size exceeding 1,000 kg in sauropodomorphs such as *Plateosauravus* (1,300 kg) and sauropods (Table 1)	Sauro.	Triassic	Increase	Bulk herbivory
4	Origin of large body size in armoured ornithischian dinosaurs (thyreophorans; Figure 1B)	Ornith.	Triassic/Jurassic	Increase	Bulk herbivory
5	Origin of small body size in heterodontosaurid ornithischians (∼0.7 kg; Table 1)	Ornith.	Triassic/Jurassic	Decrease	Specialised herbivory
6	Origin of small body size in Paraves, which has very small primitive body mass—around 1 kg (*Anchiornis*, 0.68 kg; *Microraptor*, 1.5 kg; *Archaeopteryx*, 0.97 kg (subadult))	Thero.	Jurassic	Decrease	?
7	Origin of small body size in Coelurosauria (e.g., *Ornitholestes*, 14 kg; *Zuolong*, 88 kg)	Thero.	Jurassic	Decrease	?
8	Origin of large body size in Tetanurae (from 750 kg in *Piatnitzkysaurus*).	Thero.	Jurassic	Increase	Increased macropredation
9	Origin of small body size in compsognathid coelurosaurs (*Compsognathus*, 1.6–2.3 kg)	Thero.	Jurassic	Decrease	?
10	Origin of large body size in some ceratosaurs (*Ceratosaurus*, 970 kg)	Thero.	Jurassic	Increase	Increased macropredation
11	Origin of small body size in the island dwarf sauropod *Europasaurus* (1,000 kg)	Sauro.	Jurassic	Decrease	Island dwarfing
12	Origin of large body sizes exceeding 1,000 kg in derived iguanodontians such as *Camptosaurus*	Ornith.	Jurassic	Increase	Bulk herbivory
13	Origin of large body size in the ornithuromorph birds *Yanornis* (1.5 kg) and *Yixianornis* (0.31 kg), compared with related taxa such as *Longicrusavis* (0.052 kg) and *Hongshanornis* (0.031 kg)	Thero.	Cretaceous	Increase	?Wading
14	Origin of large body size in aquatic hesperornithiform birds (e.g., *Baptornis*, 4.9 kg; *Hesperornis*, 24 kg)	Thero.	Cretaceous	Increase	Aquatic life
15	Origin of large body size in *Tianyuraptor* (20 kg) compared with other microraptoran paravians (e.g., *Graciliraptor*, 1.8 kg; *Microraptor*, 1.5 kg)	Thero.	Cretaceous	Increase	?
16	Origin of large body size in the unenlagiine dromaeosaurids *Unenlagia* (63 kg) and *Austroraptor* (519 kg)	Thero.	Cretaceous	Increase	Macropredation
17	Origin of large body size in herbivorous therizinosaurian maniraptorans (e.g., *Falcarius*, 84 kg; *Suzhousaurus*, 3,000 kg)	Thero.	Cretaceous	Increase	Bulk herbivory
18	Origin of large body size in the oviraptorosaur *Gigantoraptor* (2,000 kg)	Thero.	Cretaceous	Increase	?
19	Origin of small body size in parvicursorine alvarezsauroids.(e.g., *Parvicursor*, 0.14 kg; *Mononykus*, 4.7 kg)	Thero.	Cretaceous	Decrease	?
20	Origin of large body size in ornithomimosaurian coelurosaurs (e.g., *Shenzhousaurus*, 17 kg; *Gallimimus*, 480 kg; *Beishanlong*, 620 kg)	Thero.	Cretaceous	Increase	?Herbivory
21	Origin of large body sizes in carcharodontosaurid tetanurans (*Giganotosaurus*, 6,100 kg; *Mapusaurus*, 4,100 kg; *Carcharodontosaurus*, 3,000 kg)	Thero.	Cretaceous	Increase	Increased macropredation
22	Origin of small body size in island dwarf rhabdodontid iguanodontians (e.g., *Mochlodon vorosi*, 31 kg)	Sauro.	Cretaceous	Decrease	Island dwarfing
23	Origin of large body size in Ceratopsidae (Figure 1B)	Ornith.	Cretaceous	Increase	Bulk herbivory

Ornith., Ornithischia; Sauro., Sauropodomorpha; Thero., Theropoda.

The contrast between theropods and other dinosaurs is even greater in the Cretaceous, when no exceptional nodes occur in Sauropodomorpha, and only two in Ornithischia: at the origins of large-bodied Ceratopsidae and island dwarf rhabdodontid iguanodontians (e.g., *Mochlodon* [59]). At least nine shifts occurred during the same interval of theropod evolution, including seven in maniraptorans (Figure 3; Table 3).

## Discussion

### Niche-filling Patterns of Dinosaur Body Size Evolution

Patterns of dinosaur body size evolution are consistent with the niche-filling model of adaptive radiation [1],[4],[6]. Early dinosaurs exhibit rapid background rates of body size evolution, and a predominance of temporally rapid, order-of-magnitude shifts between body size classes in the Triassic and Early Jurassic. These shifts reflect radiation into disparate ecological niches such as bulk herbivory in large-bodied sauropodomorphs (e.g., [60]) and thyreophoran ornithischians, herbivory using a complex masticating dentition in small-bodied heterodontosaurids (e.g., [61],[62]), and increasing diversity of macropredation in large theropods (Table 3). Subsequently, rates of body size evolution decreased, suggesting saturation of coarsely defined body size niches available to dinosaurs in terrestrial ecosystems, and increasingly limited exploration of novel body size space within clades.

The early burst pattern of dinosaurian body size evolution is substantially weakened when Triassic data are excluded (Figure S1). This suggests that key innovations of Triassic dinosaurs (e.g., [63],[64]), and not the Triassic/Jurassic extinction of their competitors [37], drove the early radiation of dinosaur body sizes [34]. Indeed, phylogenetic patterns indicate that many basic ecomorphological divergences occurred well before the Triassic/Jurassic boundary.

It is not clear which innovations allowed dinosaurs to radiate [34], or whether the pattern shown here was part of a larger archosaurian radiation [65]. However, the evolution of rapid growth rates may have been important [64], especially in Sauropodomorpha [66], and the erect stance of dinosaurs and some other archosaurs [34] might have been a prerequisite for body size diversification via increased efficiency/capacity for terrestrial weight support [63].

Maniraptoran theropods are an exception to the overall pattern of declining evolutionary rates through time: exhibiting numerous instances of exceptional body size shifts, maintaining rapid evolutionary rates, and generating high ecological diversity [67],[68], including flying taxa. Although a previous study found little evidence for directional trends of body size increase in herbivorous maniraptoran clades [69], this does not conflict with our observation that some body size shifts in maniraptorans (and other coelurosaurs) coincide with the appearance of craniodental, or other, evidence for herbivory (Table 3; e.g., [67],[68],[70]).

Much of our knowledge of Late Jurassic and Early Cretaceous maniraptorans comes from a few well-sampled Chinese *Lagerstätten*, such as the Jehol biota. Without information from these exceptional deposits, we would have substantially less knowledge of divergence dates and ancestral body sizes among early maniraptorans. However, this is unlikely to bias comparisons between maniraptorans and other groups of dinosaurs for two reasons: (1) these deposits provide equally good information on the existence and affinities of small-bodied taxa in other clades, such as Ornithischia; and (2) exceptional information on early maniraptoran history should bias analyses towards finding an early burst pattern in maniraptorans. Inference of high early rates in Maniraptora would be more likely, due either to concentration of short branch durations at the base of the tree (especially using the “mbl” stratigraphic calibration method), or observation of additional body size diversity at the base of the tree that would remain undetected if sampling was poor. We cannot speculate as to the effects on our analyses of finding comparable Lagerstätten documenting early dinosaur history. However, there is currently little positive evidence that the general patterns of body size evolution documented here are artefactual.

Many stratigraphically younger dinosaurs, especially non-maniraptorans, exhibit large body size and had slow macroevolutionary rates, possibly due to scaling of generation times (e.g., [71],[72]). Scaling effects are observed across Dinosauria, but show substantial scatter (non-significant; Figure 2C) within Ornithischia and Sauropodomorpha, consistent with previous suggestions that scaling effects should be weak in dinosaurs because of the life history effects of oviparity [73]. Small dinosaurs (10–50 kg) had the highest evolutionary rates, and rates attenuated only weakly, or not at all, at sizes below 10 kg (Figure S3). This might have been key to maniraptoran diversification from small-bodied ancestors, and also explains the origins of fundamentally new body plans and ecotypes from small-bodied ancestors later in ornithischian history (Iguanodontia, Ceratopsidae; Figure 1).

### Body Size, Ecological Diversity, and Cenozoic Survival

Maniraptora includes Avialae, the only dinosaur clade to frequently break the lower body size limit around 1–3 kg seen in other dinosaurs. It is likely that more niches are available to birds (and mammals) around 100 g in mass [41],[74], so obtaining smaller body sizes might have contributed to the ecological radiation of Mesozoic birds (e.g., [27],[75]). If the K/Pg extinction event was ecologically selective, vigorous ecological diversification may have given maniraptoran lineages a greater chance of survival: Avialae was the only dinosaurian clade to survive, perhaps because of the small body sizes of its members. Although the fossil record of birds is inadequate to test hypotheses of K/Pg extinction selectivity, it is clear that smaller-sized squamates and mammals selectively survived this event [76],[77]. Therefore, our results suggest that rapid evolutionary rates within Maniraptora paved the way for a second great adaptive radiation of dinosaurs in the wake of the K/Pg extinction event: the diversification of neornithine birds [21].

### Implications for Adaptive Radiation Theory

Our findings complement recent studies of diversification rates in the avian crown group [3],[21], and suggest that birds, the most speciose class of tetrapods, arose from a long evolutionary history of continual ecological innovation. Our most striking finding is of sustained, rapid evolutionary rates on the line leading to birds (i.e., in maniraptorans) for more than 150 Ma, from the origin of dinosaurs until at least the end of the Mesozoic. Rates of evolution declined through time in most dinosaurs. However, this early burst pattern, which characterises the niche-filling model of adaptive radiation [6],[7], does not adequately describe evolution on the avian stem lineage. The recovered pattern of sustained evolutionary rates, and the repeated generation of novel ecotypes, suggests a key role for the maintenance of evolvability, the capacity for organisms to evolve, in the evolutionary success of this lineage. Evolvability might have also played a central role in the evolution of other major groups such as crustaceans [78] and actinopterygians [15], supporting its hypothesised importance in organismal evolution [79].

Rapid evolutionary rates observed during the early evolutionary history of Dinosauria, which decelerated through time in most subclades, indicate that much of the observed body size diversity of dinosaurs was generated by an early burst pattern of trait evolution. However, this pattern becomes difficult to detect when data from early dinosaurian history are not included in analyses (Figure S1), consistent with the observation that deep time data improve model inference in simulations [24]. The pruning of lineages by extinction might also overwrite the signals of ancient adaptive radiation in large neontological datasets. For example, Rabosky et al. [15] recovered slow evolutionary rates at the base of the actinopterygian tree, but the fossil record reveals substantial morphological and taxonomic diversity of extinct basal actinopterygian lineages [80],[81]. Although it has not yet been tested quantitatively, this diversity might have resulted from early rapid rates across Actinopterygii, as observed here across Dinosauria.

If our results can be generalised, they suggest that the unbalanced distribution of morphological and ecological diversity among clades results from the maintenance of rapid evolutionary rates over vast timescales in key lineages. These highly evolvable lineages may be more likely to lead to successful modern groups such as birds, whereas other lineages show declining evolutionary rates through time. Declining evolutionary rates in dinosaurian lineages off the line leading to birds indicate large-scale niche saturation. This might signal failure to keep pace with a deteriorating (biotic) environment (the Red Queen hypothesis [82],[83]), with fewer broad-scale ecological opportunities than those favouring the early radiation of dinosaurs. There is strong evidence for Red Queen effects on diversification patterns in Cenozoic terrestrial mammals [22], and it is possible that a long-term failure to exploit new opportunities characterises the major extinct radiations of deep time (and depauperate modern clades), whether or not it directly caused their extinctions.

## Materials and Methods

We used phylogenetic comparative methods to analyse rates of dinosaur body mass evolution [6],[14],[38],[39] (Appendix S1). Body mass, accompanied by qualitative observations (Table 3), was used as a general ecological descriptor. Body mass was estimated for all dinosaurs for which appropriate data were available (441 taxa; Dataset S1) using the empirical scaling relationship of limb robustness (stylopodial circumference) with body mass, derived from extant tetrapods [40] (Appendix S1). We analysed log_10_-transformed data (excluding juveniles), which represent proportional changes in body mass.

Stylopodial shaft circumferences are infrequently reported in the literature, so many were taken from our own measurements, or were calculated from shaft diameters (Appendix S1). Previous large datasets of dinosaurian masses were based on substantially less accurate methods, using the relationship between linear measurements (e.g., limb bone lengths) and volumetric models of extinct dinosaurs ([84]–[86]; reviewed by [40]).

Quantitative macroevolutionary models were tested on composite trees compiled from recent, taxon-rich cladograms of major dinosaur groups (Appendix S1; Figure S4, Figure S5, Figure S6, Figure S7). Phylogenetic uncertainty was reflected by analysing alternative topologies and randomly resolved polytomies (Appendix S1). Tip heights and branch durations were stratigraphically calibrated, and zero-length branches were “smoothed” using two methods: (1) by sharing duration equally with preceding non-zero length branches (the “equal” method [87]); and (2) by imposing a minimum branch length of 1 Ma (the “mbl” method [88]).

We used maximum-likelihood model comparison [6],[38] and “node height” test [14],[39] methods (Appendix S1) to test the prediction of the niche-filling hypothesis: that rates of morphological evolution diminish exponentially through time after an adaptive radiation [1],[2],[4]. The node height test treats standardised independent contrasts [89] as nodal estimates of evolutionary rate [39] and tests for systematic deviations from a uniform rate Brownian model, using regression against log-transformed geological age (robust regression [14],[53]). We also regressed standardised contrasts against nodal body mass estimates (a proxy for generation time and other biological processes that might influence evolutionary rates). As well as testing for a “background” model of declining evolutionary rates through time, robust regression identifies and down-weights single nodes deviating substantially from the overall pattern [14],[53]. These nodes represent substantial, temporally rapid, niche-shift events [14], following the macroecological principle that organisms in different body size classes inhabit different niches and have different energetic requirements [41]. We used lowess lines to visualise non-linear rate variation with time and body mass.

Exponentially declining rates of evolution through time, predicted by the niche-filling model of adaptive radiation [1]–[3], were also tested by comparing the fit of an early burst model [6],[7] with other commonly used models: Brownian motion, directional evolution (“trend”), the Ornstein–Uhlenbeck model of evolution attracted to an optimum value, and stasis (“white noise”) [38],[56],[90] (Appendix S1). Explicit mathematical models of trait evolution on our phylogenies were fitted using the R packages GEIGER version 1.99–3 [91] and OUwie version 1.33 [55] (for Ornstein–Uhlenbeck (OU) models only), and compared using AICc [92],[93]. Unlike GEIGER, OUwie allows estimation of a trait optimum (θ) that is distinct from the root value (Z_0_) in OU models. Values from GEIGER and OUwie are directly comparable: identical log likelihood, AICc, and parameter estimates are obtained for test datasets when fitting models implemented in both packages (Brownian motion in all instances; and OU models when θ = Z_0_ for ultrametric trees); although note that comparable standard error values entered to the OUwie function of OUwie 1.33 are the square of those entered to the fitContinuous function of Geiger 1.99–3. The algorithm used to fit OU models in GEIGER 1.99–3 is inappropriate for non-ultrametric trees (personal communication, Graham Slater to R. Benson, December 2013). This problem is specific to OU models implemented by GEIGER 1.99–3, and does not affect the other models that we tested. GEIGER 1.99–3 fits models of trait evolution using independent contrasts, after rescaling the branch lengths of the phylogenetic tree according to the model considered [7]. For all models, except the OU model in the case of non-ultrametric trees, the covariance between two taxa i and j can be written as a function of the path length s_ij_ shared between the two taxa (e.g., [6],[7]). The tree can thus easily be rescaled by applying this function to the height of each node before computing independent contrasts. In the case of the OU model, the covariance between two taxa i and j is a function of both the shared (pre-divergence) portion of their phylogenetic history and the non-shared (post-divergence) portion [54]. In the case of an ultrametric tree, the non-shared portion can also be written as a function of s_ij_ (it is simply the total height T of the tree, minus s_ij_ [90],[94]), and the corresponding scaling function can be applied to the tree (this is what is performed in GEIGER 1.99.3). However, in the case of a non-ultrametric tree, the post-divergence portion of the covariance cannot be written as a function of s_ij_, so there is no straightforward scaling function to apply. Instead, it is necessary to fit the model by maximum likelihood after computing the variance–covariance matrix. This is what is implemented in OUwie, and now in GEIGER 2.0 (personal communication, Josef Uyeda to R. Benson, January 2014).

Our data and analytical scripts are available at DRYAD [95].

## Supporting Information

Figure S1 **Node height test for early burst of rates of dinosaur body mass evolution excluding Triassic nodes.** Results of robust regression of evolutionary rate on node age: (A) slope; (B) p-value. Dashed lines occur at zero (A) and 0.05 (B); 1 = Dinosauria; 2 = Ornithischia; 3 = Sauropodomorpha; 4 = Theropoda; and 5 = Maniraptora.
(TIF)

Figure S2 **AICc weights of maximum likelihood models using different trees and time calibration methods.** AICc weights are shown for early burst (1–5), trend (6), and Ornstein–Uhlenbeck (7) models. (A) Trees including the Yates topology for non-sauropodan sauropodomorphs (Figure S6), and calibrated using the “equal” method (*Materials and Methods*). (B) Trees including the Upchurch topology for non-sauropodan sauropodomorphs (Figure S7), and calibrated using the “equal” method. (C) Trees including the Yates topology for non-sauropodan sauropodomorphs, and calibrated using the “mbl” method (*Materials and Methods*). (D) Trees including the Upchurch topology for non-sauropodan sauropodomorphs, and calibrated using the “mbl” method.
(TIF)

Figure S3 **A possible non-linear relationship between macroevolutionary rate and nodal body mass.** (A) Based on one phylogeny calibrated using the “equal” method (*Materials and Methods*). (B) Based on one phylogeny calibrated using the “mbl” method (*Materials and Methods*). The (solid) lowess lines suggests that rates decrease with body mass above ∼10−50 kg, but might also decline with a shallower gradient below ∼10−50 kg. The dashed lines show the fitted linear robust regressions.
(TIF)

Figure S4 **Composite tree of ornithischian dinosaur relationships used in the present study.** Polytomies were resolved randomly prior to analyses. Details of tree construction are given in Appendix S1.
(TIF)

Figure S5 **Composite tree of theropod dinosaur relationships used in the present study.** Polytomies were resolved randomly prior to analyses. Details of tree construction are given in Appendix S1.
(TIF)

Figure S6 **Composite tree of sauropodomorph relationships used in the present study, using the Yates topology for non-sauropodans.** Polytomies were resolved randomly prior to analyses. Details of tree construction are given in Appendix S1.
(TIF)

Figure S7 **Composite tree of sauropodomorph relationships used in the present study, using the Upchurch et al. topology for non-sauropodans.** Polytomies were resolved randomly prior to analyses. Details of tree construction are given in Appendix S1.
(TIF)

Table S1 **Summary of ordinary least-squares regression relationships between femoral and humeral anteroposterior and mediolateral shaft diameters for groups.** N, sample size; R^2^, coefficient of determination.
(DOC)

Table S2 **Proportions of phylogenies for which data simulated under a constant rate Brownian motion model generated robust regression slopes (node height test) shallower than those observed in the data in fewer than 0.05, 0.10, 0.15, or 0.20 of simulated datasets.** Analyses excluding Maniraptora are shaded in grey, and results based only on phylogenies calibrated to stratigraphy different methods (see *Materials and Methods*) are additionally presented for Dinosauria. ** indicates cases in which all phylogenies reject the constant rate model at the specified threshold, and * indicates cases in which most phylogenies reject the constant rate model at the specified threshold. Values should not be regarded as p-values, but generally concur with the p-values of our robust regression fits (Figure 2B).
(DOC)

Appendix S1 Additional methods and results.
(DOC)

Dataset S1 Complete dataset and mass estimates.
(XLS)

## Abbreviations

AICc: Akaike's information criterion for finite sample sizes
Ma: million years
mbl: minimum branch length
