# Correction: Rates of Dinosaur Body Mass Evolution Indicate 170 Million Years of Sustained Ecological Innovation on the Avian Stem Lineage

**DOI:** 10.1371/journal.pbio.1001896

**Published:** 2014-06-02

**Authors:** The PLOS Biology Staff

## Notice of Republication

This article was republished on May 19, 2014, to replace Figure 3 with a version which is available under the Creative Commons CC0 public domain dedication. The figure caption was also amended to clarify this is the case. Please download this article again to view the correct version.
