# Supplementary figures and images for: Rates of Dinosaur Body Mass Evolution Indicate 170 Million Years of Sustained Ecological Innovation on the Avian Stem Lineage
Source: PLoS Biol. 2014 May 6;12(5):e1001853. doi: 10.1371/journal.pbio.1001853 (PMC4011683; doi:10.1371/journal.pbio.1001853)

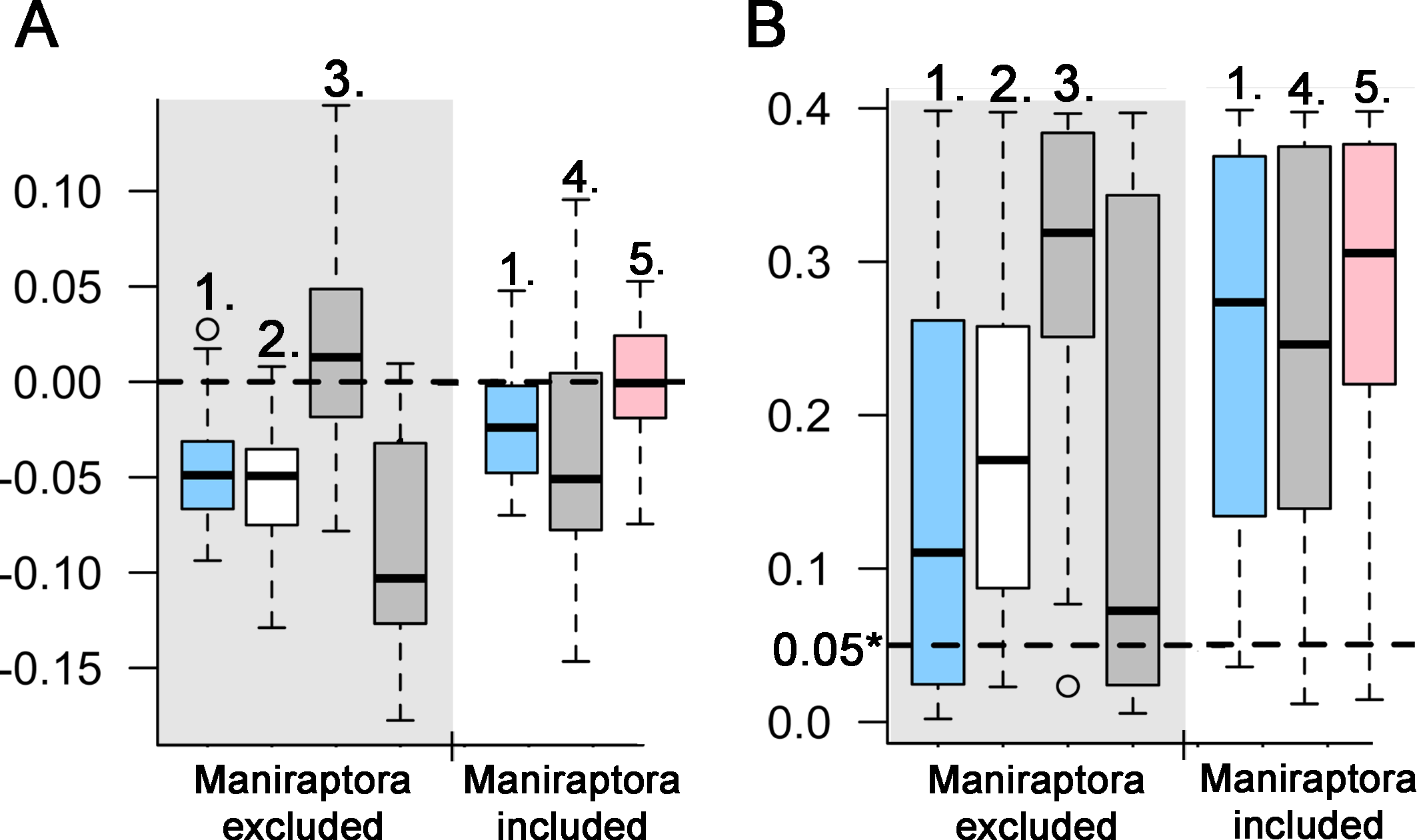

Supplement: Figure S1 — Node height test for early burst of rates of dinosaur body mass evolution excluding Triassic nodes. Results of robust regression of evolutionary rate on node age: (A) slope; (B) p-value. Dashed lines occur at zero (A) and 0.05 (B); 1 = Dinosauria; 2 = Ornithischia; 3 = Sauropodomorpha; 4 = Theropoda; and 5 = Maniraptora. (TIF) [file pbio.1001853.s001.tif]

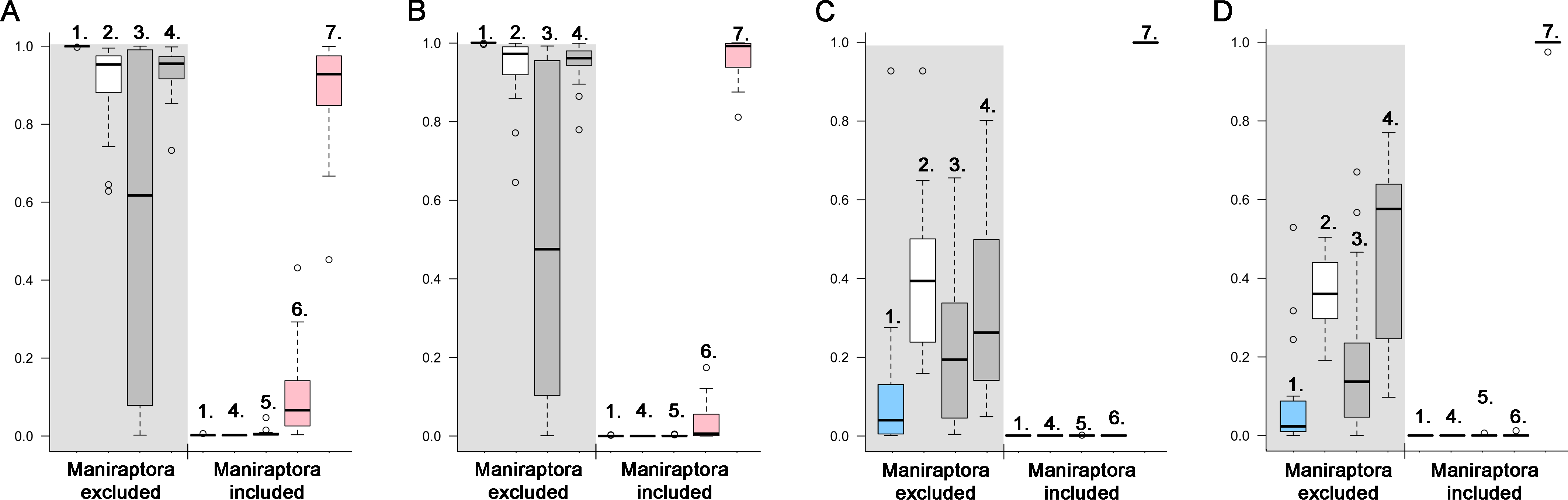

Supplement: Figure S2 — AICc weights of maximum likelihood models using different trees and time calibration methods. AICc weights are shown for early burst (1–5), trend (6), and Ornstein–Uhlenbeck (7) models. (A) Trees including the Yates topology for non-sauropodan sauropodomorphs (Figure S6), and calibrated using the “equal” method ( Materials and Methods ). (B) Trees including the Upchurch topology for non-sauropodan sauropodomorphs (Figure S7), and calibrated using the “equal” method. (C) Trees including the Yates topology for non-sauropodan sauropodomorphs, and calibrated using the “mbl” method ( Materials and Methods ). (D) Trees including the Upchurch topology for non-sauropodan sauropodomorphs, and calibrated using the “mbl” method. (TIF) [file pbio.1001853.s002.tif]

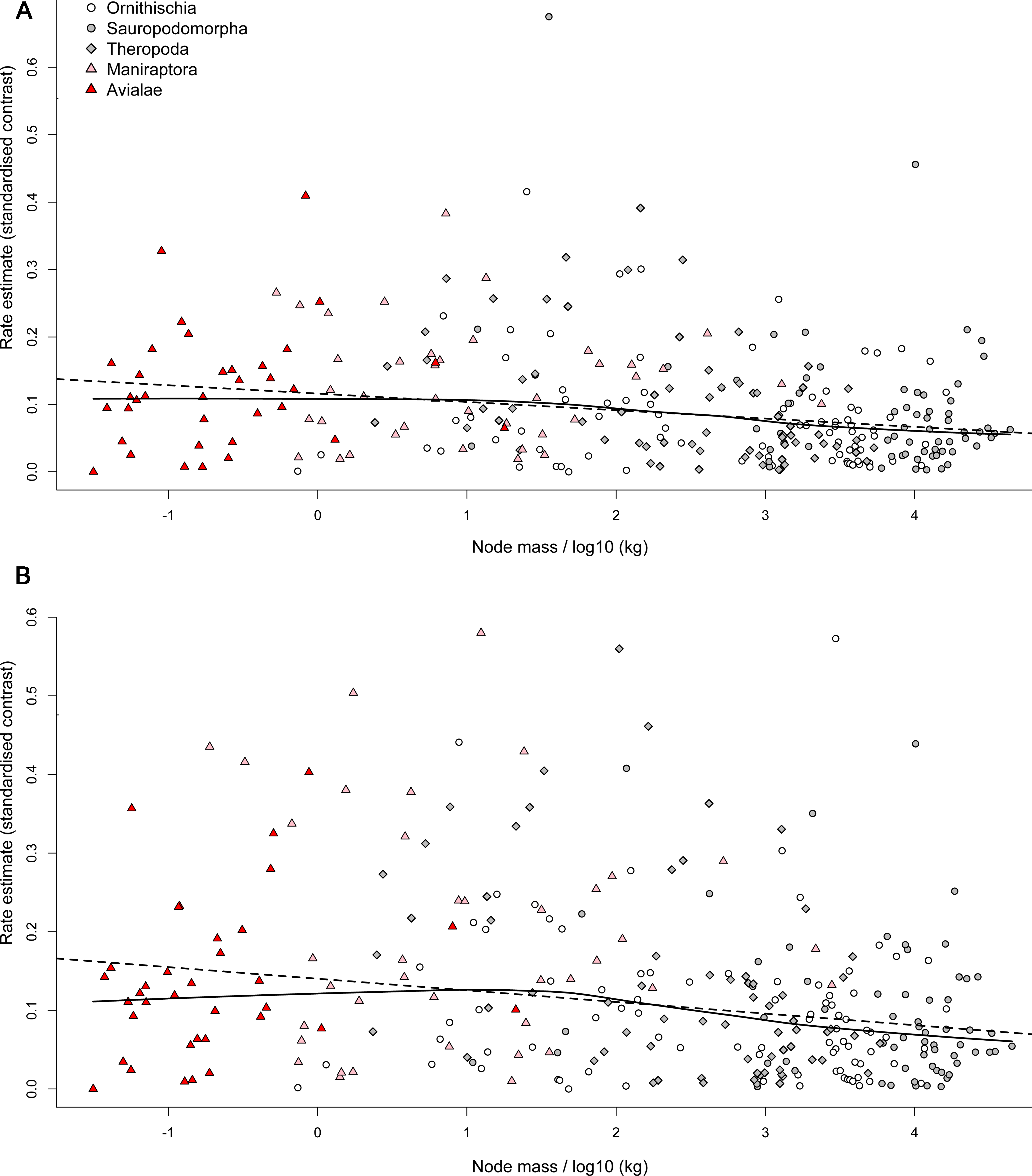

Supplement: Figure S3 — A possible non-linear relationship between macroevolutionary rate and nodal body mass. (A) Based on one phylogeny calibrated using the “equal” method ( Materials and Methods ). (B) Based on one phylogeny calibrated using the “mbl” method ( Materials and Methods ). The (solid) lowess lines suggests that rates decrease with body mass above ∼10−50 kg, but might also decline with a shallower gradient below ∼10−50 kg. The dashed lines show the fitted linear robust regressions. (TIF) [file pbio.1001853.s003.tif]

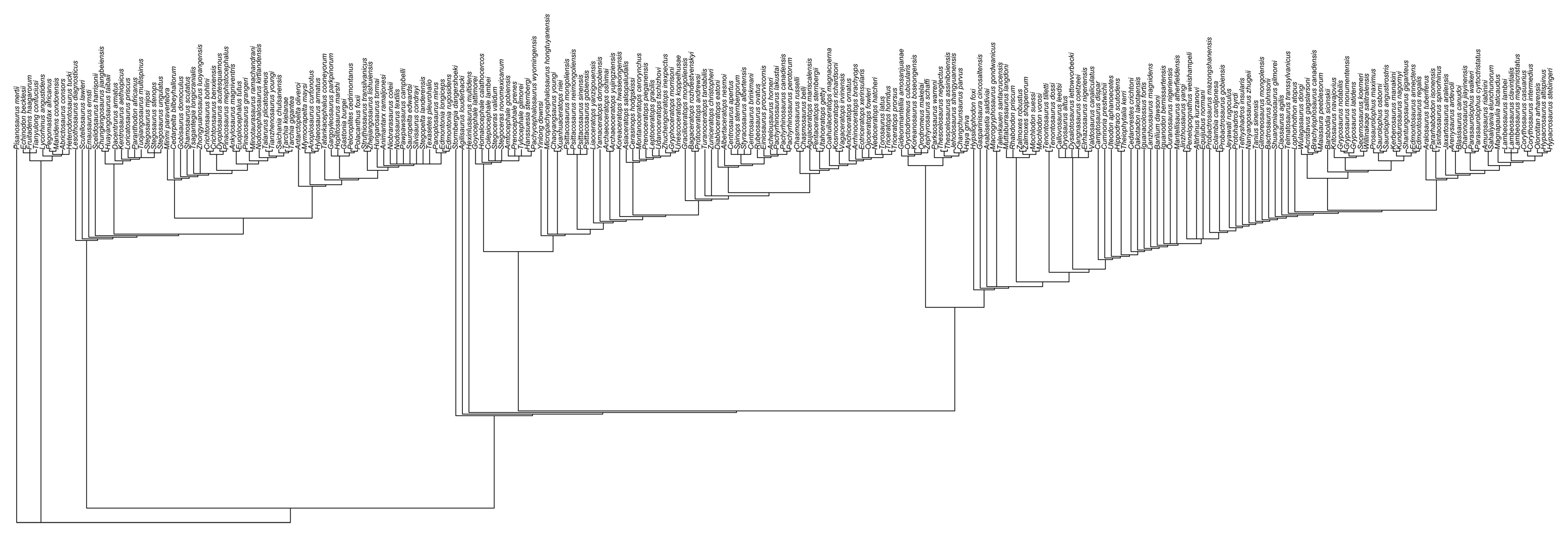

Supplement: Figure S4 — Composite tree of ornithischian dinosaur relationships used in the present study. Polytomies were resolved randomly prior to analyses. Details of tree construction are given in Appendix S1. (TIF) [file pbio.1001853.s004.tif]

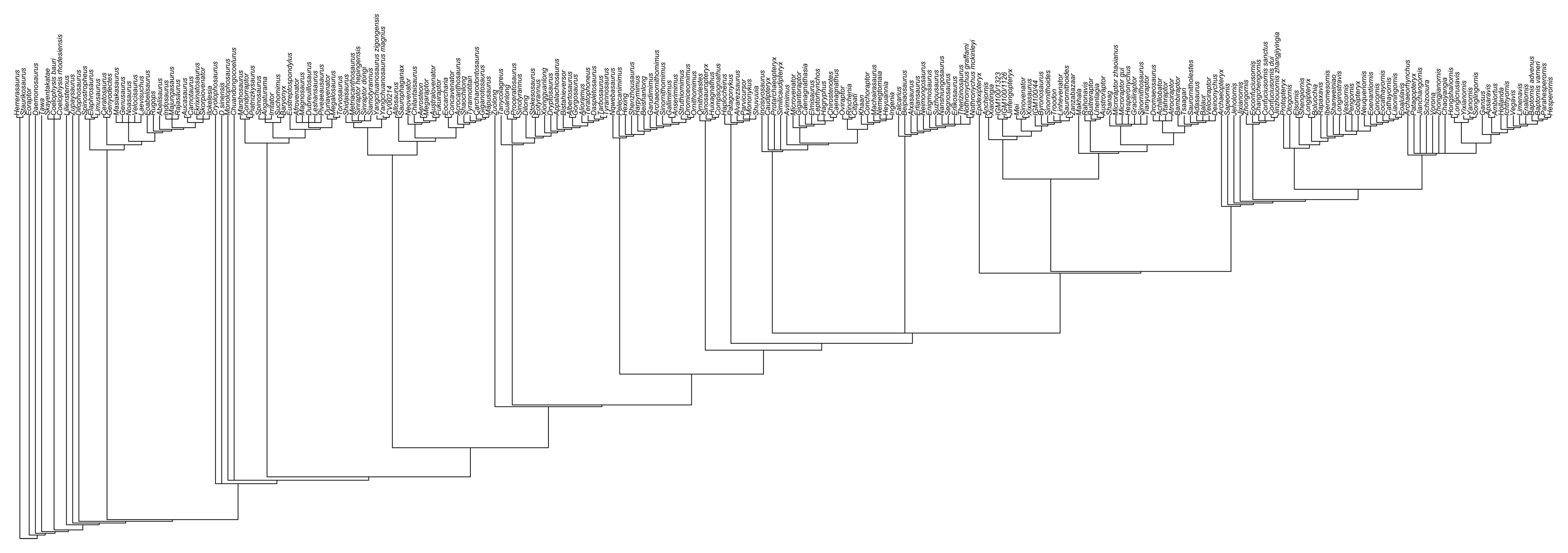

Supplement: Figure S5 — Composite tree of theropod dinosaur relationships used in the present study. Polytomies were resolved randomly prior to analyses. Details of tree construction are given in Appendix S1. (TIF) [file pbio.1001853.s005.tif]

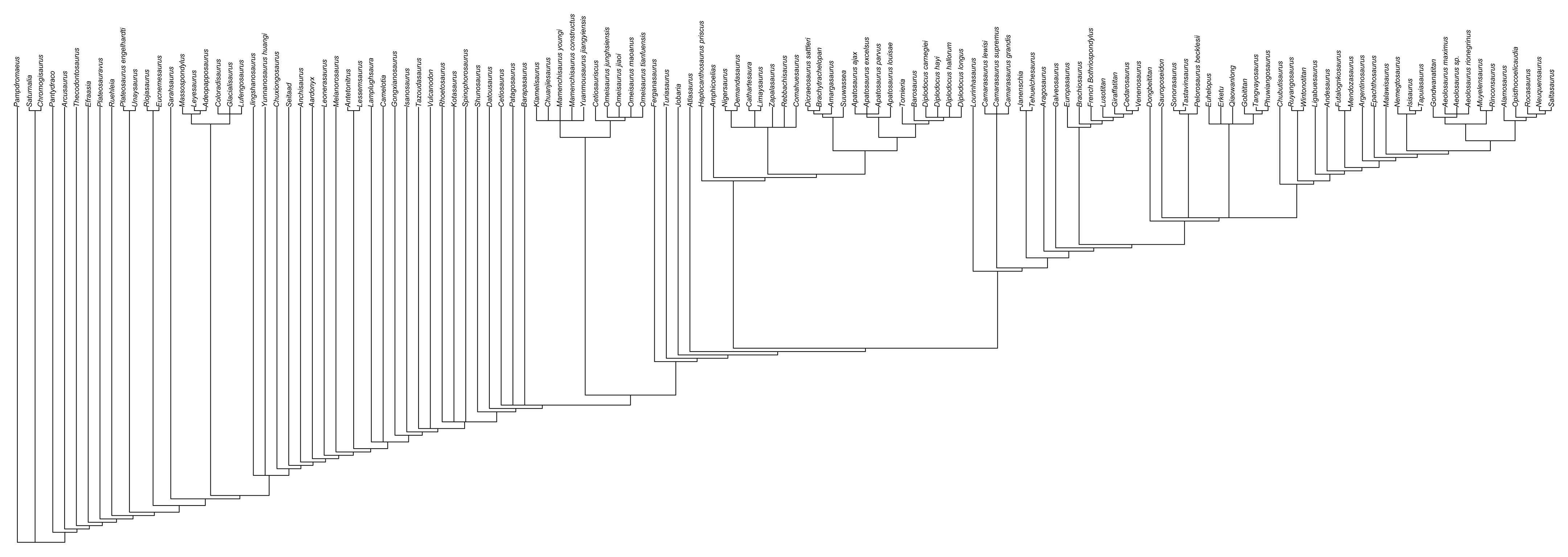

Supplement: Figure S6 — Composite tree of sauropodomorph relationships used in the present study, using the Yates topology for non-sauropodans. Polytomies were resolved randomly prior to analyses. Details of tree construction are given in Appendix S1. (TIF) [file pbio.1001853.s006.tif]

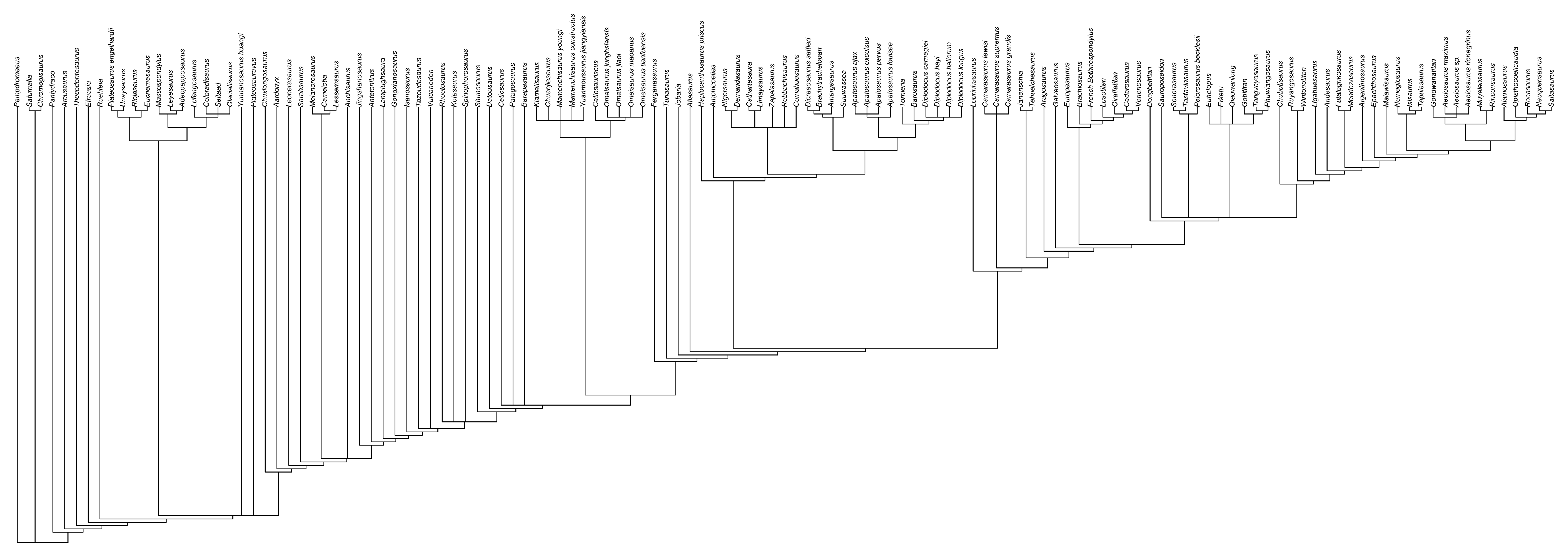

Supplement: Figure S7 — Composite tree of sauropodomorph relationships used in the present study, using the Upchurch et al. topology for non-sauropodans. Polytomies were resolved randomly prior to analyses. Details of tree construction are given in Appendix S1. (TIF) [file pbio.1001853.s007.tif]
