# Supplementary material for: Rates of Dinosaur Body Mass Evolution Indicate 170 Million Years of Sustained Ecological Innovation on the Avian Stem Lineage
Source: PLoS Biol. 2014 May 6;12(5):e1001853. doi: 10.1371/journal.pbio.1001853 (PMC4011683; doi:10.1371/journal.pbio.1001853)
Supplement: Table S1 — Summary of ordinary least-squares regression relationships between femoral and humeral anteroposterior and mediolateral shaft diameters for groups. N, sample size; R2, coefficient of determination. (DOC) [file pbio.1001853.s008.doc]

| **Group** | **Femoral diameters** | | | **Humeral diameters** | | |
| --- | --- | --- | --- | --- | --- | --- |
|  | **N** | **R^2^** | ***p*-value** | **N** | **R^2^** | ***p*-value** |
| Allosauroidea | 10 | 0.957 | 0.000 | NA | NA | NA |
| Alvarezsauroidea | 5 | 0.987 | 0.001 | NA | NA | NA |
| Ankylosauria | 9 | 0.615 | 0.012 | 9 | 0.332 | 0.104 |
| Avialae | 11 | 0.979 | 0.000 | NA | NA | NA |
| ‘basal Coelurosauria’ | 5 | 0.982 | 0.001 | NA | NA | NA |
| ‘basal Theropoda’ | 8 | 0.920 | 0.000 | NA | NA | NA |
| ‘basal Ceratopsia’ | 5 | 0.986 | 0.001 | 3 | 0.977 | 0.097 |
| Ceratopsidae | 6 | 0.478 | 0.128 | 6 | 0.872 | 0.006 |
| Ceratosauria | 7 | 0.944 | 0.000 | NA | NA | NA |
| Diplodocoidea | 12 | 0.592 | 0.003 | 8 | 0.602 | 0.024 |
| Dromaeosauridae | 6 | 0.995 | 0.000 | NA | NA | NA |
| ‘basal Eusauropoda’ | 5 | 0.979 | 0.001 | 9 | 0.912 | 0.000 |
| Hadrosauroidea | 10 | 0.265 | 0.128 | 9 | 0.261 | 0.160 |
| ‘basal Iguanodontia’ | 15 | 0.925 | 0.000 | 7 | 0.987 | 0.000 |
| ‘basal Macronaria’ | NA | NA | NA | 4 | 0.822 | 0.093 |
| Megalosauroidea | 5 | 0.876 | 0.019 | NA | NA | NA |
| ‘basal Ornithischia’ | 15 | 0.924 | 0.000 | 6 | 0.922 | 0.002 |
| Ornithomimosauria | 5 | 0.772 | 0.050 | NA | NA | NA |
| Oviraptorosauria | 3 | 0.832 | 0.269 | NA | NA | NA |
| Pachycephalosauria | NA | NA | NA | NA | NA | NA |
| ‘basal Sauropoda’ | 7 | 0.077 | 0.546 | 4 | 0.075 | 0.725 |
| ‘basal Sauropodomorpha’ | 11 | 0.943 | 0.000 | 9 | 0.918 | 0.000 |
| Stegosauria | 5 | 0.778 | 0.048 | 6 | 0.883 | 0.005 |
| Therizinosauria | 3 | 0.979 | 0.093 | NA | NA | NA |
| ‘basal Thyreophora’ | NA | NA | NA | NA | NA | NA |
| Titanosauria | 11 | 0.867 | 0.000 | 14 | 0.775 | 0.000 |
| ‘basal’ Titanosauriformes | 6 | 0.832 | 0.011 | 4 | 0.940 | 0.030 |
| Troodontidae | 3 | 0.998 | 0.030 | NA | NA | NA |
| Tyrannosauroidea | 4 | 0.975 | 0.013 | NA | NA | NA |
