# Supplementary material for: Rates of Dinosaur Body Mass Evolution Indicate 170 Million Years of Sustained Ecological Innovation on the Avian Stem Lineage
Source: PLoS Biol. 2014 May 6;12(5):e1001853. doi: 10.1371/journal.pbio.1001853 (PMC4011683; doi:10.1371/journal.pbio.1001853)
Supplement: Table S2 — Proportions of phylogenies for which data simulated under a constant rate Brownian motion model generated robust regression slopes (node height test) shallower than those observed in the data in fewer than 0.05, 0.10, 0.15, or 0.20 of simulated datasets. Analyses excluding Maniraptora are shaded in grey, and results based only on phylogenies calibrated to stratigraphy different methods (see Materials and Methods ) are additionally presented for Dinosauria. ** indicates cases in which all phylogenies reject the constant rate model at the specified threshold, and * indicates cases in which most phylogenies reject the constant rate model at the specified threshold. Values should not be regarded as p-values, but generally concur with the p-values of our robust regression fits (Figure 2B). (DOC) [file pbio.1001853.s009.doc]

|  | **0.05** | **0.10** | **0.15** | **0.20** |
| --- | --- | --- | --- | --- |
| **Dinosauria (non-maniraptoran) (‘equal’)** | 0.93* | 0.97* | 1.00** | 1.00** |
| **Dinosauria (non-maniraptoran) (‘mbl’)** | 0.47 | 0.73* | 0.83* | 0.90* |
| **Ornithischia** | 0.55* | 0.63* | 0.75* | 0.82* |
| **Sauropodomorpha** | 0.20 | 0.33 | 0.48 | 0.55* |
| **Theropoda (non-maniraptoran)** | 0.85* | 0.99* | 1.00** | 1.00** |
| **Dinosauria (‘equal’ calibration)** | 0.37 | 0.77* | 0.90* | 0.90* |
| **Dinosauria (‘mbl’ calibration)** | 0.03 | 0.17 | 0.17 | 0.23 |
| **Theropoda** | 0.47 | 0.62* | 0.75* | 0.82* |
| **Maniraptora** | 0.05 | 0.08 | 0.15 | 0.18 |
