# Supplementary material for: Rates of Dinosaur Body Mass Evolution Indicate 170 Million Years of Sustained Ecological Innovation on the Avian Stem Lineage
Source: PLoS Biol. 2014 May 6;12(5):e1001853. doi: 10.1371/journal.pbio.1001853 (PMC4011683; doi:10.1371/journal.pbio.1001853)
Supplement: Appendix S1 — Additional methods and results. (DOC) [file pbio.1001853.s010.doc]

**Appendix S1**

**Additional methods and results**

Rates of Dinosaur Body Mass Evolution Indicate 170 Million Years of Sustained Ecological Innovation on the Avian Stem Lineage.

Roger B. J. Benson, Nicolás E. Campione, Matthew T. Carrano, Philip D. Mannion, Corwin Sullivan, Paul Upchurch & David C. Evans

Dinosaur mass estimates

Based on our own measurements and the literature, we compiled a species level dataset of dinosaur limb bone measurements for use in mass estimation, based on well-constrained scaling relationships between body mass and the minimum circumferences of the mass-supporting stylopodials (Campione & Evans 2012). Macroevolutionary analyses used log_10_-transformed body mass estimates.

The power of stylopodial circumferences to predict live mass was first observed by Anderson et al. (1985). Using a large dataset of extant reptiles and mammals, Campione & Evans (2012) showed that the combined humeral and femoral circumference represents a robust proxy for estimating body mass that is largely independent of phylogenetic history, gait, and limb posture in quadrupeds. Because many dinosaurs were bipedal, a consistent method using only the femoral circumference was required. Consequently, we use an equation that corrects for the systematic bias generated when measuring the circumference of two bones (i.e. a quadruped) versus one bone (i.e. a biped). This systematic bias can be used to generate a coefficient (2^0.5^) that is integrated into the quadrupedal equation of Campione & Evans (2012), permitting its application to bipedal taxa, and yields mass estimates that are substantially similar to the widely used equation of Christiansen & Farina (2004). The coefficient for bipedal taxa was derived geometrically, following the principal that shaft circumference is a proxy for shaft cross-sectional area, a measure of capacity for supporting weight. It corrects for the fact that the total shaft cross-sectional area for two bones (humerus and femur) has a different relationship with the summed shaft circumferences than does that of a single bone (the femur). Both our bipedal and quadrupedal equations are based on the same baseline extant dataset, and therefore represent consistent and related models on which to generate mass estimates in dinosaurs without the need to invoke outside sources of information.

Masses in kilograms were estimated using femoral (FC) and humeral (HC) circumferences and these equations:

[1] mass = 10^a^/1000

Where, for bipedal taxa:

[2] a_bipedal_ = 2.749*log_10_(FC * 2^0.5^) – 1.104

And for quadrupedal taxa:

[3] a_quadrupedal_ = 2.749*log_10_(FC + HC) – 1.104

Dinosaurs were considered to be primitively bipedal. Thus, the masses of theropods (including Mesozoic birds), many ornithischians, and most non-sauropodan sauropodomorphs were estimated using equation [2]. Within Sauropodomorpha, the clade comprising Melanorosaurus, Camelotia, and Lessemsaurus plus Sauropoda was considered to be quadrupedal [e.g. Yates et al. 2010]. Riojasauridae (Riojasaurus + Eucnemesaurus), and the clade of Jingshanosaurus and all taxa more closely related to Sauropoda than to Jingshanosaurus (e.g. Aardonyx), were considered to be at least facultatively quadrupedal (Yates et al. 2010; Bonaparte & Pumares 1995; Bonnan 2003; Yates & Kitching 2003; Yates 2007), so we estimated their masses using both equations [2] and [3], and examined how this affected our results.

Three ornithischian clades were also considered to be quadrupedal: (1) Thyreophora; (2) most ceratopsians (Leptoceratopsidae and all taxa more closely related to Ceratopsidae except Cerasinops and Udanoceratops were treated as certainly quadrupedal; neoceratopsians outside this clade, Cerasinops, and Udanoceratops, were treated as facultatively quadrupedal) (Chinnery 2004, 2007; Zhao et al. 2013); and (3) Hadrosauroidea and some other iguanodontians (Mantellisaurus, Iguanodon, and all taxa more closely related to Hadrosauroidea were treated as certainly quadrupedal; iguanodontians outside this clade were treated as facultatively quadrupedal) (Norman 1980, 1986; Maidment et al. 2012; Maidment & Barrett in press). We also performed analyses in which all iguanodontians were considered to be bipedal. All these possibilities yielded near-identical analytical results.

Our data enable 94 mass estimates of bipedal dinosaurs (or 100 estimates treating facultative quadrupeds as bipeds), and 156 mass estimates of quadrupedal dinosaurs (or 141 estimates treating facultative quadrupeds as quadrupeds). However, after estimation of circumferences from diameters (see Estimating circumferences from diameters, below), 137–146 bipedal mass estimates and 304–280 quadrupedal mass estimates were possible. Thus 441–426 dinosaurian masses were estimated from our data, of which adult mass estimates for 310 taxa were included in our largest composite tree (see Time-scaled phylogeny, below). Skeletal maturity was assessed from published histological studies (e.g., Lee & Werning 2008; Benton et al. 2010; Erickson et al. 2006, 2009a,b, 2010; Osi et al. 2012; Werning 2012) and qualitative indicators such as the fusion of neurocentral and neurocranial sutures. Only mass estimates of adult individuals were used in our analyses.

Previous recent dinosaur mass estimates were based on volumetric computer models (e.g., Henderson 1999; Bates et al. 2009; Sellers et al. 2013), which are time-intensive to construct and require the preservation of near-complete skeletons. They therefore cannot provide data for macroevolutionary study at large sample sizes. Some authors estimated live masses of incomplete specimens using the scaling relationships between linear measurements such as femoral length, and volumetric model mass estimates (Christiansen & Farina 2004; Seebacher 2001; Mazzetta et al. 2004; O’Gorman & Hone 2012; Zanno & Mackovicky 2013). However, linear measurements such as limb lengths are poor estimators of mass in extant tetrapods (Campione & Evans 2012), and this was presumably also true of extinct taxa. Furthermore, scaling relationships between limb lengths and volumetric mass estimates of extinct taxa have not accounted for errors in the volumetric models, which have wide but often unquantified error bars (Bates et al. 2009; Hutchinson et al. 2011).

**Estimating circumferences from diameters**

To estimate femoral circumferences from observed diameters, we parsed our data into a set of 29 groups, including paraphyletic grades (denoted ‘basal’). These were intended to represent approximate ‘body plan’ groupings that should have similar relationships between humeral and femoral shaft diameters and their circumferences, a hypothesis that was tested using regression:

'basal Theropoda'

Ceratosauria

Megalosauroidea

Allosauroidea

'basal Coelurosauria'

Tyrannosauroidea

Ornithomimosauria

Alvarezsauroidea

Therizinosauria

Oviraptorosauria

Dromaeosauridae

Troodontidae

Avialae

'basal Ornithischia'

‘basal Thyreophora’

Stegosauria

Ankylosauria

‘basal Ceratopsia’

Ceratopsidae

Pachycephalosauria

‘basal Iguanodontia’

Hadrosauroidea

'basal Sauropodomorpha'

‘basal Sauropoda’

‘basal Eusauropoda’

Diplodocoidea

‘basal Macronaria’

‘basal Titanosauriformes’

Titanosauria

For each of the femur and humerus, each group contained some taxa for which the minimum shaft circumference and both its anteroposterior and mediolateral diameters were known, some taxa for which only a subset of these measurements were known, and some taxa for which none of these were known (for example, if the bone was not preserved in a specimen of that taxon). We estimated femoral circumferences for taxa in which at least one diameter was known by taking the following steps:

(1) We estimated the ordinary least squares regression equation of anteroposterior shaft diameter on mediolateral shaft diameter, and mediolateral shaft diameter on anteroposterior shaft diameter for each group in which both measurements were known in at least three taxa.

(2) For groups in which a significant (*p* < 0.05) regression relationship existed between the diameters, we used those relationships to predict the second diameter measurement for taxa in which only one diameter measurement was known. In general, bipedal groups with sufficient sample sizes had well-constrained relationships between the diameters of their mass-supporting stylopodials, but the relationship was weaker in some quadrupedal groups, especially Ceratopsidae, Hadrosauroidea and Sauropoda, suggesting they exhibit more variable eccentricity (Table S1).

(3) We used equation [4] below to convert pairs of diameters (d_ml_ = mediolateral diameter; d_ap_ = anteroposterior diameter) into circumferences, assuming that the bone shaft has an oval cross-section (circumference_oval_):

[4] circumference_oval_ = pi * ((3 * (d_ml_ + d_ap_)) - (((3 * d_ml_ + d_ap_)*(d_ml_ + 3 * d_ap_))^0.5^))

(4) Measured shaft circumference was regressed through the origin on circumference_oval_ for each group. All R^2^ values exceeded 0.985 and the slopes of the regression lines (ranging from 0.92–1.10) were used as correction factors to translate circumference_oval_ into an estimate of the true shaft circumference for taxa in which a measured shaft circumference was not known. Some groups had too little data to estimate a correction factor. Thus, the factor for Dromaeosauridae was used for Avialae and Alvarezsauroidea, the factor for Titanosauriformes was used for Macronaria, the factor for Eusauropoda was used for Sauropoda, the factor for basal Ornithischia was used for Pachycephalosauria, and the median factor for all groups was used for Therizinosauria.

Time-scaled phylogeny

Composite trees of 614–622 taxa were constructed using large, recent cladistic datasets for major clades of theropods (Kobayashi & Barsbold 2005; Choiniere et al. 2010, 2012; Makovicky et al. 2010; Zanno 2010; Sues et al. 2011; Carrano et al. 2012; Pol & Rauhut 2012; Turner et al. 2012; Brusatte & Benson 2013; Longrich et al. 2013; O’Connor & Zhou 2013), sauropodomorphs (Wilson 2002; Upchurch et al. 2004, 2007; González-Riga et al. 2009; Santucci & Arruda-Campos 2011; Sekiya 2011; Whitlock 2011; Carballido et al. 2012; D’Emic 2012; Apaldetti et al. 2013; Mannion et al. 2013), and ornithischians (Butler et al. 2008; Maidment et al. 2008; Evans 2010; Prieto-Marquez et al. 2012; Sampson et al. 2010; Godefroit et al. 2012; McDonald 2012; Sereno 2012; Thompson et al. 2012). The full tree file is available at DRYAD (http://datadryad.org/resource/doi:10.5061/dryad.gr1qp), and the versions included in our analyses are illustrated in Figs S4–S7.

Our trees included approximately 116 unresolved nodes, reflecting lack of phylogenetic consensus for some groups and taxa. These nodes were randomly resolved prior to analyses, and results were consistent among multiple random resolutions, suggesting they are robust to phylogenetic uncertainty. We also compared results based on incongruent topologies for non-sauropodan sauropodomorphs (based on Upchurch et al. [2007] and Yates [2007]).

Ten non-dinosaurian dinosauromorphs (six silesaurids, Marasuchus and three lagerpetonids) were included in our tree to help calibrate the durations of basal dinosaur divergences without relying on an arbitrarily chosen ‘root length’. Branch durations were estimated based on the stratigraphic ages of taxa using the timePaleoPhy function of the R package paleotree version 1.4 (Bapst 2012), incorporating uncertainty by drawing taxon ages randomly from a uniform distribution between their maximum and minimum possible ages at substage resolution. Zero length branches were lengthened using two methods: (1) by equally sharing duration from the immediately basal non-zero length branch (Brusatte et al. 2008) using the ‘equal’ option of timePaleoPhy; and (2) by imposing a minimum branch duration of 1 Ma using the ‘mbl’ option of timePaleoPhy (Laurin 2004). We also examined trees calibrated with a minimum branch duration of 2 Ma, but these were not used as they contained implausibly ancient divergences within Dinosauria (Middle–Late Permian: 268–278 Ma). Juveniles and subadult specimens, non-dinosaurian dinosauromorphs, and taxa for which mass estimates were not available, were deleted from the tree following stratigraphic calibration. Both node calibration methods yielded similar analytical results (see below and main text).

Major dinosaurian divergences were generally dated to the Early Triassic or earlier: the Ornithischia-Saurischia split was estimated around (1) 244–249 Ma (‘equal’ method) or (2) 240–253 Ma (‘mbl’ 1 Ma). Younger dates were obtained for basal splits in Ornithischia ((1) 233–236 Ma; (2) 232–238 Ma), Sauropodomorpha ((1) 227–240 Ma; (2) 232–246 Ma), and Theropoda ((1) 238–243 Ma; (2) 232–239 Ma). Dates from ‘equal’ and ‘mbl’ 1 Ma, are reasonable given the first fossil appearances of ornithischians, sauropodomorphs and theropods in the late Carnian [~230 Ma; e.g., Bonaparte 1976; Sereno et al. 1993; Langer et al. 1999], and possible occurrences of Dinosauria as old as 244 Ma (Nesbitt et al. 2012). The ‘equal’ method generally gives older divergence dates for Theropoda. The ‘mbl’ method gives older dates for Sauropodomorpha because of their taxonomic abundance in the Late Triassic. These divergence dates extend back to the Early Triassic and are probably overestimates, which is likely to bias our results against detecting an early burst pattern in Sauropodomorpha using ‘mbl’ trees.

Maximum likelihood models — method

Explicit mathematical models of trait evolution were fit to comparative data on a phylogenetic tree with branch lengths using Maximum likelihood (Pagel 1999) in the R packages Geiger version 1.99-3 (Harmon et al. 2008) and OUwie version 1.33 (Beaulieu et al. 2012, for Ornstein-Uhlenbeck models only). These models quantify the tempo and mode of macroevolutionary change, and the best model can be selected using Akaike’s information criterion for finite sample sizes (AICc; Sugiura 1978; Burnham & Anderson 2004). Models do not describe reality in its totality. Furthermore, individual models might capture different aspects of trait evolution when multiple factors are important in reality. Nonetheless, they are useful in summarising the major features of trait macroevolution, and establishing their relative importance.

Declining rates of evolution through time, predicted by the niche-filling model of adaptive radiation (Simpson 1953; Schluter 2000), can be tested by comparing the fit of an ‘early burst’ (‘b’, below) or accelerating/decelerating (ACDC) model (Harmon et al. 2010; Blomberg et al. 2003) with other models (Pagel 1999; Butler & King 2004; Hunt & Carrano 2010). Candidate models are listed below.

(a) Brownian motion (‘BM’) is equivalent to a random walk along phylogenetic lineages. This results in pairwise differences among taxa that have an expectation of zero, and covariance proportional to the duration of their shared ancestry (e.g., Cunningham et al. 1998: box 3), scaled by a global Brownian variance parameter (β; Martins & Hansen 1997: equation 6a (their γ)). Brownian variance is a measure of evolutionary rate when Brownian-like non-directional macroevolution occurs (Felsenstein 1973, 1985; Hunt 2012). In total, two parameters of Brownian motion models are estimated by maximising likelihood, β and the trait value at the root of the tree (Z_0_). Several other models listed below described non-Brownian evolution by introducing an additional parameter (early burst, trend, and Ornstein-Uhlenbeck models).

(b) In the ‘early burst’ (‘EB’) model, Brownian variance (evolutionary rate) takes a value at the root (β_0_), but changes through time. The parameter r describes the pattern of exponential rate change through time (r = 0 corresponds to Brownian motion; r < 0 yields exponentially declining rates through time; Harmon et al. 2010). This is a ‘one-tailed’ case of the ACDC model (in which rates can either accelerate or decelerate through time; Blomberg et al. 2003).

(c) Directional evolution (‘trend’) is equivalent to Brownian motion with non-zero expectation, scaled according to time since the root by the parameter μ (mean step length). For example, when μ is positive, trait values generally increase through time (e.g., Pagel 2002; Hunt & Carrano 2010), and μ=0 corresponds to Brownian motion.

(d) The Ornstein-Uhlenbeck (‘OU’) model of evolution constrained towards an optimum value (Martins & Hansen 1997; Hansen 1997). This is modified from Brownian motion by the inclusion of a scaling parameter α, specifying the strength of attraction towards an optimum θ, which can equal the root value (Z_0_), or be estimated as a separate parameter (Beaulieu et al. 2012). These parameters result in a ‘rubber band’ effect that attracts trait values asymptotically to θ (Martins & Hansen 1997; Hansen 1997). When trait values are approximately equal to θ, stasis-like evolution occurs with a variance of β and a mean of θ (see model ‘e’ below). Unlike the other models described, the OU model was fit using the R package OUwie, which allows estimation of θ separately from Z_0_ (Beaulieu et al. 2012). The OUwie algorithm calculates expected variances along each branch of a phylogenetic tree under an OU model, and is therefore appropriate for non-ultrametric trees, such as those analysed here (J. Beaulieu, pers. comm., January 2014). OUwie also allows multiple macroevolutionary regimes to be specified, each with different values of α, θ, and β. However, this is not relevant to our specific hypothesis tests, and was not attempted.

(e) Stasis (white noise) in which each tip value is estimated independently from a normal distribution with two parameters (the mean and standard deviation).

An intraspecific error of 0.135 log_10_(kg) was assumed in all analyses. This value is the standard error of mass estimates based on stylopodial circumferences for tetrapods (Campione & Evans 2012), and is likely to dwarf other sources of interspecific error.

Maximum likelihood models — results

Early burst models generally receive the greatest AICc weights for analyses excluding maniraptorans (non-maniraptoran Dinosauria, Ornithischia, Sauropodomorpha, and non-maniraptoran Theropoda) (Table 2; Fig. S2). This is especially true for trees dated using the ‘equal’ method, for which early burst is overwhelmingly supported compared to all other models. Some variation in model fits is evident on trees dated using the ‘mbl’ method, although note that phylogenies dated using the mbl method likely overestimate divergence dates among early sauropodomorphs, biasing our analyses of Sauropodomorpha and Dinosauria as a whole against detection of early burst (see *Time-scaled phylogeny*, above).

When mbl time-calibration is used: (1) Ornstein-Uhlenbeck models can have better fit than early burst models for Dinosauria when including maniraptorans; (2) Ornstein-Uhlenbeck and trend models have non-negligible fit to ornithischian body size evolution, although early burst is still the best model for most phylogenies; and (3) Ornstein-Uhlenbeck models have non-negligible fit to sauropodomorph body size evolution, although early burst is still the best model for most phylogenies.

The occasional fit of Ornstein-Uhlenbeck models indicates that dinosaurian body mass evolution is characterised by attraction to large body size optima in Ornithischia, and especially in Sauropodomorpha. However, this does not invalidate the strong fit of early burst models, or our node height test results, as these models capture different aspects of dinosaurian body size evolution.

Early burst in maniraptorans (and in Dinosauria and Theropoda when including maniraptorans) receives vanishingly little support (other than in Dinosauria calibrated using the ‘equal method’). Instead, maniraptoran body mass evolution is best explained by trend or Ornstein-Uhlenbeck models (Fig. S2), consistent with the occurrence of smallest body sizes for most maniraptoran clades during the Early Cretaceous.

Node height test

The node height test (Freckleton & Harvey 2006) uses the absolute values of phylogenetically independent contrasts (PIC; Felsenstein 1985; Garland et al. 1992; here calculated using the R package ape 3.0-8; Paradis et al. 2004) on a time-calibrated tree, scaled to their expected variances (i.e., standardised contrasts), as point estimates of Brownian variance, or evolutionary rate (Felsenstein 1973, 1985; Hunt 2012). This works on the principal that PIC is a computationally simple way to fit a Brownian motion model (Garland & Ives 2000; Blomberg et al. 2012). However, the node height test does not assume Brownian motion. Instead, it is a test for deviations from the predictions of Brownian motion. Specifically, the prediction that contrast magnitudes do not correlate with ‘node heights’, the estimated node ages relative to the root of the tree (Freckleton & Harvey 2006). The niche-filling model of adaptive radiation predicts exponentially declining rates through time (e.g., Harmon et al. 2010; Slater & Pennell in press), so we used log_10_-transformed node ages to represent an exponential decrease in evolutionary rate through time.

Slater & Pennell (in press) suggested the use of Maximum likelihood-type (M-type) robust regression in the node height test, and showed using simulations that this increased method performance (i.e. decreased Type II error) compared to comparisons of AICc values among maximum-likelihood models (described above). M-type robust regression is an iterative procedure that down-weights observations with large residual values to minimise the influence of outliers on the regression slope (Huber 1973). Thus, single nodes with exceptionally high or low contrasts do not bias the overall model fit (unlike in current maximum-likelihood model fitting approaches; Slater & Pennell in press). These exceptional nodes can easily be identified by their weights (< 1; Slater & Pennell in press).

We examined whether the slopes of our robust regressions could be generated by a uniform, single rate Brownian motion model applied to each of our phylogenies by simulating 1000 datasets for each of our analyses using the sim.char function of Geiger (Harmon et al. 2008), setting the ancestral body mass and Brownian variance equal to those inferred from model fitting. These simulations essentially confirmed the significance (p-values) from our robust regressions (Table S2).

It is also possible that a correlation between body mass and evolutionary rates, combined with higher average body mass in stratigraphically younger dinosaur species could drive an apparent decrease in evolutionary rates through time. For example, larger taxa have longer generation times and therefore slower time-scaled microevolutionary rates (Gingerich 2001, 2009; Evans et al. 2012; Okie et al. 2013), or might be under greater physical or ecological constraints than small-bodied taxa. We tested this possibility by conducting robust regression of absolute contrasts on nodal (log-transformed) body mass, estimated using square change parsimony. The data distribution for two time-scaled phylogenies is shown in Figure S3, and discussed in the text. Results from alternative phylogenies are similar.

**References**

Anderson JF, Hall-Martin A, Russell DA. 1985. Long-bone circumference and weight in mammals, birds and dinosaurs. Journal of Zoology 207, 53–61.

Apaldetti C, Pol D, Yates A. 2013. The postcranial anatomy of *Coloradisaurus brevis* (Dinosauria: Sauropodomorpha) from the Late Triassic and its phylogenetic implications. Palaeontology 56, 277–301.

Bapst DW. 2012. paleotree: an R package for paleontological and phylogenetic analyses of evolution. Methods in Ecology and Evolution 3, 803–807.

Bates KT, Falkingham PL, Breithaupt BH, Hodgetts D, Sellers WI, Manning PL. 2009. How big was ‘Big Al’? Quantifying the effect of soft tissue and osteological unknowns on mass predictions for *Allosaurus* (Dinosauria: Theropoda). Palaeontologica Electronica 12(3), 14A.

Beaulieau JM, Jhuwueng D-C, Boettiger C, O’Meara BC. 2012. Modelling stablizing selection: expanding the Ornstein-Uhlenbeck model of adaptive evolution. Evolution 66-8, 2369–2383.

Benton MJ, Csiki Z, Grigorescu D, Redelstorff R, Sander PM, Stein K, Weishampel DB. 2010. Dinosaurs and the island rule: the dwarfed dinosaurs from Hateg Island Palaeogeography, Palaeolcimatology, Palaeoecology 293, 438–454.

Blomberg SP, Garland T, Ives AR. 2003. Testing for phylogenetic signal in comparative data: behavioural traits are more labile. Evolution 57, 717–745.

Blomberg SP, Lefevre JG, Wells JA, Waterhouse M. 2012. Independent contrasts and PGLS regression estimators are equivalent. Systematic Biology 61, 382–391.

Bonaparte JF. 1976. *Pisanosaurus mertii* Casamiquela and the origin of the Ornithischia. Journal of Paleontology 50, 808–820.

Bonaparte JF, Pumares JA. 1995. Notas sobre primer craneo de *Riojasaurus incertus* (Dinosauria, Prosauropoda, Melanorosauridae) del Triasico Superior de La Rioja, Argentina. Ameghiniana 32, 341–349.

Bonnan MF. 2003. The evolution of manus shape in sauropod dinosaurs: implications for functional morphology, forelimb orientation, and phylogeny. Journal of Vertebrate Paleontology 23, 595–613.

Brusatte SL, Benson RBJ. 2013 The systematics of Late Jurassic tyrannosauroids (Dinosauria: Theropoda) from Europe and North America. Acta Palaeontologica Polonica (doi:10.4202/app.2011.0141).

Brusatte SL, Benton MJ, Ruta M, Lloyd GT. 2008. Superiority, competition, and opportunism in the evolutionary radiation of dinosaurs. Science 321, 1485–1487.

Burnham KP, Anderson DR. 2004. Model selection and multimodel inference. Springer, New York, 488 pp.

Butler MA, King AA. 2004. Phylogenetic comparative analysis: a modelling approach for adaptive radiation. American Naturalist 164, 683-695.

Butler RJ, Upchurch P, Norman DB. 2008. The phylogeny of the ornithischian dinosaurs. Journal of Systematic Palaeontology 6, 1–40.

Campione NE, Evans DC. 2012. A universal scaling relationship between body mass and proximal limb bone dimensions in quadrupedal terrestrial tetrapods. BMC Biology 10(60), 1–21.

Carballido JL, Salgado L, Pol D, Canudo JI, Garrido A. 2012. A new basal rebbachisaurid (Sauropoda, Diplodocoidea) from the Early Cretaceous of the Neuquén Basin; evolution and biogeography of the group. Historical Biology 24: 631–654.

Carrano MT, Benson RBJ, Sampson SD. 2012 The phylogeny of Tetanurae (Dinosauria: Theropoda). Journal of Systematic Palaeontology 10, 211–300.

Chinnery B. 2004. Morphometric analysis of evolutionary trends in the ceratopsian postcranial skeleton. Journal of Vertebrate Paleontology 24, 591–609.

Chinnery BJ, Horner JR. 2007. A new neoceratopsian dinosaur linking North American and Asian taxa. Journal of Vertebrate Paleontology 27, 625–641.

Choiniere JN, Xu X, Clark JM, Forster CA, Guo Y, Han F. 2010 A basal alvarezsauroid theropod from the early Late Jurassic of Xinjiang, China. Science 327, 571–574.

Choiniere JN, Forster C, de Klerk WJ. 2012 New information on *Nqwebasaurus thwazi*, a coelurosaurian theropod from the Early Cretaceous Kirkwood Formation in South Africa. Journal of African Earth Sciences 71–72, 1–17.

Christiansen P, Farina RA. 2004. Mass prediction in theropod dinosaurs. Historical Biology 16, 85–92.

Cunningham CW, Omland KE, Oakley TH. 1998. Reconstructing ancestral character states: a critical reappraisal. Trends in Ecology and Evolution 13, 361–366.

D’Emic MD. 2012. The early evolution of titanosauriform sauropod dinosaurs. Zoological Journal of the Linnean Society 166, 624–671.

Erickson GM, Currie PJ, Inouye BD, Winn AA. 2006. Tyrannosaur life tables: an example of nonavian dinosaur population biology. Science 313, 213–217.

Erickson GM, Rauhut OWM, Zhou Z-H, Turner AH, Inouye BD, Hu D-Y, Norell MA 2009a. Was dinosaurian physiology inherited by birds? Reconciling slow growth in *Archaeopteryx*. PLoS ONE 4, e7390.

Erickson GM, Makovicky PJ, Inouye BD, Zhou C-F, Gao K. 2009b. Life table for *Psittacoaurus lujiatunensis*: the first glimpse into ornithischian dinosaur population biology. Anatomical Record 292, 1514–1521.

Erickson GM, Currie PJ, Inouye BD, Winn AA. 2010. A revised life table and survivorship curve for *Albertosaurus sarcophagus* based ont eh Dry Island mass death assemblage. Canadian Journal of Earth Sciences 47, 1269–1275.

Evans AR, Jones D, Boyer AG, Brown JH, Costa DP, Ernest SKM, Fitzgerald EMG, Fortelius M, Gittleman JL, Hamilton MJ, Harding LE, Lintulaakso K, Lyons SK, Okie JG, Saarinen JJ, Sibly RM, Smith FA, Stephens PR, Theodor JM, Huehn MD. 2012. The maximum rate of mammal evolution. Proceedings of that National Academy of Sciences of the USA 109, 4187–4190.

Evans DC. 2010. Cranial anatomy and systematics of *Hypacrosaurus altispinus*, and a comparative analysis of skull growth in lambeosaurine hadrosaurids (Dinosauria: Ornithischia). Zoological Journal of the Linnean Society 159, 398–434.

Felsenstein J. 1973. Maximum-likelihood estimation of evolutionary trees from continuous characters. American Journal of Human Genetics 25, 471–492.

Felsenstein J. 1985. Phylogenies and the comparative method. American Naturalist 125, 1–15.

Freckleton RP, Harvey PH. 2006. Detecting non-Brownian trait evolution in adaptive radiations. PLoS Biology 4(11), e373.

Garland T, Ives AR. 2000. Using the past to predict the present: confidence intervals for regression equations in phylogenetic comparative methods. American Naturalist 155, 346–364.

Garland T, Harvey PH, Ives AR. 1992. Procedures for the analysis of comparative data using phylogenetically independent contrasts. Systematic Biology 41, 18–32.

Gingerich PD. 2001. Rates of evolution on the time scale of the evolutionary process. Genetica 112–113, 127–144.

Gingerich PD. 2009. Rates of evolution. Annual Reviews in Ecology and Systematics 40, 657–675.

Godefroit P, Bolotsky YL, Lauters P. 2012. A New Saurolophine Dinosaur from the Latest Cretaceous of Far Eastern Russia. PLoS ONE 7, e36849.

González-Riga BJ, Previtera E, Pirrone CA. 2009. *Malarguesaurus florenciae* gen. et sp. nov., a new titanosauriform (Dinosauria, Sauropoda) from the Upper Cretaceous of Mendoza, Argentina. Cretaceous Research 30, 135–148.

Hansen TF. 1997. Stabilizing selection and the comparative analysis of adaptation. Evolution 51, 1341–1351.

Harmon LJ, Weir JT, Brock CD, Glor RE, Challenger W. 2008. GEIGER: investigating evolutionary radiations. Bioinformatics 24, 129–131.

Harmon LJ, Losos JB, Davies TJ, Gillespie RG, Gittleman JL, Jennings WB, Kozak KH, McPeek MA, Moreno-Roark F, Near TJ, Purvis A, Ricklefs RE, Schulter D, Schulte JA 2^nd^, Seehausen O, Sidlauskas BL, Torres-Carvajal O, Weir JT, Mooers AO. 2010. Early bursts of body size and shape evolution are rare in comparative data. Evolution 64, 2385–2396.

Henderson DM .1999. Estimating the mass and centers of mass of extinct animals by 3D mathematical slicing. Paleobiology 25:88–106.

Huber PJ. 1973. Robust regression: asymptotics, conjectures and Monte Carlo. Annals of Statistics 1, 799–821.

Hunt G. 2012. Measuring rates of phenotypic evolution and the inseparability of tempo and mode. Paleobiology 38, 351–373.

Hunt G, Carrano MT. 2010. Models and methods for analyzing phenotypic evolution in lineages and clades. Special Papers of the Paleontological Society 16, 245–269.

Hutchinson JR, Bates KT, Molnar J, Allen V, Makovicky PJ. 2011. A computational analysis of limb and body dimensions in *Tyrannosaurus rex* with implications for locomotion, ontogeny and growth. PLoS ONE 6(10): e26037.

Kobayashi Y, Barsbold R. 2005 Anatomy of *Harpymimus okladnikovi* Barsbold and Perle 1984 (Dinosauria; Theropoda) of Mongolia. In *The Carnivorous Dinosaurs* (ed. K. Carpenter), pp. 97–126. Bloomington, Indiana, USA: Indiana University Press.

Langer MC, Abdala F, Richter M, Benton MJ. 1999. A sauropodomoprh dinosaur from the Upper Triassic (Carnian) of southern Brazil. Comptes Rendus de l’Academie des Sciences Series IIA Earth and Planetary Science 329, 511–517.

Laurin M. 2004. Evolution of body size, Cope’s rule and the origin of amniotes. Systematic Biology 53, 594–622.

Lee AH, Werning S. 2008. Sexual maturity in growing dinosaurs does not fit reptilian growth models. Proceedings of that National Academy of Sciences of the USA 105, 582–587.

Longrich NR, Barnes K, Clark S, Millar L. 2013. Caenagnathidae from the Upper Campanian Aguja Formation of west Texas, and a revision of the Caenagnathidae. Bulletin of the Peabody Museum of Natural History 54, 23–49.

Maidment SCR, Barrett PM. in press. Osteological correlates for quadrupedality in ornithischian dinosaurs Acta Palaeontologica Polonica (doi: 10.4202/app.2012.0065).

Maidment SCR, Norman DB, Barrett PM, Upchurch P. 2008 Systematics and phylogeny of Stegosauria (Dinosauria: Ornithischia) Journal of Systematic Palaeontology 6, 367–407.

Maidment SCR, Linton DH, Upchurch P, Barrett PM. 2012. Limb-one scaling indicates diverse stance and gait in quadrupedal ornithischian dinosaurs. PLoS ONE 7, e36904.

Makovicky PJ, Li D, Gao K-Q, Lewin M, Erickson GM, Norell MA. 2010 A giant ornithomimosaur from the Early Cretaceous of China. Proceedings of the Royal Society B 277, 191–198.

Mannion PD, Upchurch P, Barnes RN, Mateus O. 2013. Osteology of the Late Jurassic Portuguese sauropod dinosaur Lusotitan atalaiensis (Macronaria) and the evolutionary history of basal titanosauriforms. Journal of Systematic Palaeontology 168, 98–206.

Martins EP, Hansen TF. 1997. Phylogenies and the comparative method: a general approach to incorporating phylogenetic information into the analysis of interspecific data. American Naturalist 149, 646–667.

Mazzetta GV, Christiansen P, Fariña RA. 2004. Giants and bizarres: body size of some southern South American Cretaceous dinosaurs. Historical Biology 2004, 1–13.

McDonald AT. 2012. Phylogeny of basal iguanodonts (Dinosauria: Ornithischia): an update. PLoS ONE 7, e36745.

Nesbitt SJ, Barrett PM, Werning S, Sidor CA, Charig AJ. 2012. The oldest dinosaur? A Middle Triassic dinosauriform from Tanzania. Biology Letters 9, 20120949.

Norman DB. 1980. On the ornithischian dinosaur Iguanodon bernissatensis from the Lower Cretaceous of Bernissart (Belgium). Mémoires de l'Institut Royal des Sciences Naturelles de Belgique 178, 1–103.

Norman DB. 1986. On the anatomy of Iguanodon atherfieldensis (Ornithischia: Ornithopoda). Bulletin de l'Institut Royal des Sciences Naturelle de Belgique: Sciences de la Terre 56, 281–372.

O’Connor JK, Zhou Z-H. 2013 A redescription of *Chaoyangia beishanensis* (Aves) and a comprehensive phylogeny of Mesozoic birds. Journal of Systematic Palaeontology.

O’Gorman EJ, Hone DWE. 2012. Body size distribution of the dinosaurs. PLOS ONE 7(12), e51925.

Okie JG, Boyer AG, Brown JH, Costa DP, Ernest SKM, Evans AR, Fortelius M, Gittleman JL, Hamilton MJ, Harding LE, Lintulaakso K, Lyons SK, Saatinen JJ, Smith FA, Stephens PR, Theodor J, Uhen MD, Sibly RM. 2013. Effects of allometry, productivity and lifestyle on rates and limits of body size evolution. Proceedings of the Royal Society B 280, 20131007.

Ősi A, Prondvai E, Butler R, Weishampel DB. 2012. Phylogeny, histology and inferred body size evolution in a new rhabdodontid dinosaur from the Late Cretaceous of Hungary. PLoS ONE 7, e44318.

Pagel M. 1999. Inferring the historical patterns of biological evolution. Nature 401, 877–884.

Pagel M. 2002. Modelling the evolution of continuously varying characters on phylogenetic trees: the case of hominid cranial capacity. Pp. 269–286 in (MacLeod N, Forey PL, eds), Morphology, Shape and Phylogeny. Taylor & Francis, London.

Paradis E, Claude J, Strimmer K. 2004. APE: analysis of phylogenetics and evolution in R language. Bioinformatics 20, 289–290.

Pol D, Rauhut OWM. 2012 A Middle Jurassic abelisaurid from Patagonia and the early diversification of theropod dinosaurs. Proceedings of the Royal Society B 279, 3170–3175.

Prieto-Márquez A, Chiappe LM, Joshi SH. 2012. The Lambeosaurine Dinosaur *Magnapaulia laticaudus* from the Late Cretaceous of Baja California, Northwestern Mexico. PLoS ONE 7, e38207.

Sampson SD, Loewen MA, Farke AA, Roberts EM, Forster CA, Smith JA, Titus AL. 2010. New horned dinosaurs from Utah provide evidence for intracontinental dinosaur endemism. PLoS ONE 5(9): e12292.

Santucci RM, Arruda-Campos AC. 2011. A new sauropod (Macronaria, Titanosauria) from the Adamantina Formation, Bauru Group, Upper Cretaceous of Brazil and the phylogenetic relationships of Aeolosaurini. Zootaxa 3085, 1–33.

Schluter D. 2000. The ecology of adaptive radiation. Oxford University Press, Oxford, 288 pp.

Seebacher F. 2001. A new method to calculate allometric length-mass relationships of dinosaurs. Journal of Vertebrate Paleontology 21, 51–60.

Sekiya T. 2011. Re-examination of *Chuanjiesaurus anaensis* (Dinosauria:

Sauropoda) from the Middle Jurassic Chuanjie Formation, Lufeng County, Yunnan Province, southwest China. Memoir of the Fukui Prefectural Dinosaur Museum 10: 1–54.

Sellers WI, Hepworth-Bell J, Falkingham PL, Bates KT, Brassey CA, Egerton VM, Manning PL. 2013. Minimum convex hull mass estimations of complete mounted skeletons. Biology Letters 8, 842–845.

Sereno PC. 2012. Taxonomy, morphology, masticatory function and phylogeny of heterodontosaurid dinosaurs. ZooKeys 226, 1–225.

Sereno PC, Forster CA, Rogers RR, Moneta AM. 1993. Primitive dinosaur skeleton from Argentina and the early evolution of Dinosauria. Nature 361, 64–66.

Simpson GG. 1953. The major features of evolution. Columbia University Press, New York, 434 pp.

Slater GJ, Pennell MW. in press. Robust regression and posterior predictive simulation increase power to detect early bursts of trait evolution. Systematic Biology.

Sues, H-D, Nesbitt SJ, Berman DS, Henrici AC. 2011 A late-surviving basal theropod dinosaur from the latest Triassic of North America. Proceedings of the Royal Society B 278, 3459–3464.

Sugiura N. 1978. Further analysis of the data by Akaike’s Information Criterion and the Finite Corrections. Comunications in Statistics, Theory and Methods 7, 13–26.

Thompson RS, Parish JC, Maidment SCR, Barrett PM. 2012. Phylogeny of the ankylosaurian dinosaurs (Ornithischia: Thyreophora) Journal of Systematic Palaeontology 10, 301–312.

Turner AH, Makovicky PJ, Norell MA. 2012 A review of dromaeosaurid systematics and paravian phylogeny. Bulletin of the American Museum of Natural History 371, 1–206.

Upchurch, P., Barrett, P. M. and Dodson, P. 2004. Sauropoda. In: Weishampel DB, Dodson P, Osmólska H, eds. The Dinosauria, 2nd edition. Berkeley: University of California Press, 259–324.

Upchurch P, Barrett PM, Galton PM. 2007. The phylogenetic relationships of basal sauropodomorphs: implications for the origin of sauropods. Special Papers in Palaeontology 77, 57–90.

Werning S. 2012. The ontogenetic osteohistology of *Tenontosaurus tilletti* PLOS ONE 7(3), e33539.

Whitlock JA. 2011. A phylogenetic analysis of Diplodocoidea (Saurischia: Sauropoda). Zoological Journal of the Linnean Society 161, 872–915.

Wilson JA 2002. Sauropod dinosaur phylogeny: critique and cladistic analysis. Zoological Journal of the Linnean Society 136, 217–276.

Yates AM. 2007. The first complete skull of the Triassic dinosaur *Melanorosaurus* Haughton (Sauropodomorpha: Anchisauria). Special Papers in Palaeontology 77, 9–55.

Yates AM, Kitching JW. 2003. The earliest known sauropod dinosaur and the first steps towards sauropod locomotion. Proceedings of the Royal Society B 270, 1753–1758.

Yates AM, Bonnan MF, Neveling J, Chinsamy A, Blackbeard MG. 2010. A new transitional sauropodomorph dinosaur from the Early Jurassic of South Africa and the evolution of sauropod feeding and quadrupedalism. Proceedings of the Royal Society B 277, 787–794.

Zanno LE. 2010 A taxonomic and phylogenetic re-evaluation of Therizinosauria (Dinosauria: Maniraptora). Journal of Systematic Palaeontology 8, 503–543.

Zanno LE, Makovicky PJ. 2013. No evidence for directional evolution of body mass in herbivorous theropod dinosaurs. Proceedings of the Royal Society B 280, 20122526.

Zhao Q, Benton MJ, Sullivan C, Sander PM, Xu X. 2013. Histology and postural change during the growth of the ceratopsian dinosaur *Psittacosaurus lujiatunensis*. Nature Communications 4, 2079.
